# ROS-responsive liposomes as an inhaled drug delivery nanoplatform for idiopathic pulmonary fibrosis treatment via Nrf2 signaling

**DOI:** 10.1186/s12951-022-01435-4

**Published:** 2022-05-06

**Authors:** Junzhao Liu, Zuohong Wu, Yadong Liu, Zhu Zhan, Liping Yang, Can Wang, Qinqin Jiang, Haitao Ran, Pan Li, Zhigang Wang

**Affiliations:** 1 Department of Ultrasound, Chongqing Key Laboratory of Ultrasound Molecular Imaging, The Second Affiliated Hospital of Chongqing Medical University, Chongqing, China; 2 Department of Respiratory and Critical Care Medicine, Chongqing Traditional Chinese Medicine Hospital, Chongqing, China; 3 Key Laboratory of Laboratory Medical Diagnostics Designated by Chinese Ministry of Education, Chongqing Medical University, Chongqing, China; 4 Institute of Ultrasound Imaging, The Second Affiliated Hospital of Chongqing Medical University, Chongqing, China

**Keywords:** Idiopathic pulmonary fibrosis, ROS-responsive liposome, Inhaled drug delivery, Dimethyl fumarate, Nrf2

## Abstract

**Background:**

Idiopathic pulmonary fibrosis (IPF) is a progressive fibrotic disease with pathophysiological characteristics of transforming growth factor-β (TGF-β), and reactive oxygen species (ROS)-induced excessive fibroblast-to-myofibroblast transition and extracellular matrix deposition. Macrophages are closely involved in the development of fibrosis. Nuclear factor erythroid 2 related factor 2 (Nrf2) is a key molecule regulating ROS and TGF-β expression. Therefore, Nrf2 signaling modulation might be a promising therapy for fibrosis. The inhalation-based drug delivery can reduce systemic side effects and improve therapeutic effects, and is currently receiving increasing attention, but direct inhaled drugs are easily cleared and difficult to exert their efficacy. Therefore, we aimed to design a ROS-responsive liposome for the Nrf2 agonist dimethyl fumarate (DMF) delivery in the fibrotic lung. Moreover, we explored its therapeutic effect on pulmonary fibrosis and macrophage activation.

**Results:**

We synthesized DMF-loaded ROS-responsive DSPE-TK-PEG@DMF liposomes (DTP@DMF NPs). DTP@DMF NPs had suitable size and negative zeta potential and excellent capability to rapidly release DMF in a high-ROS environment. We found that macrophage accumulation and polarization were closely related to fibrosis development, while DTP@DMF NPs could attenuate macrophage activity and fibrosis in mice. RAW264.7 and NIH-3T3 cells coculture revealed that DTP@DMF NPs could promote Nrf2 and downstream heme oxygenase-1 (HO-1) expression and suppress TGF-β and ROS production in macrophages, thereby reducing fibroblast-to-myofibroblast transition and collagen production by NIH-3T3 cells. In vivo experiments confirmed the above findings. Compared with direct DMF instillation, DTP@DMF NPs treatment presented enhanced antifibrotic effect. DTP@DMF NPs also had a prolonged residence time in the lung as well as excellent biocompatibility.

**Conclusions:**

DTP@DMF NPs can reduce macrophage-mediated fibroblast-to-myofibroblast transition and extracellular matrix deposition to attenuate lung fibrosis by upregulating Nrf2 signaling. This ROS-responsive liposome is clinically promising as an ideal delivery system for inhaled drug delivery.

**Graphical Abstract:**

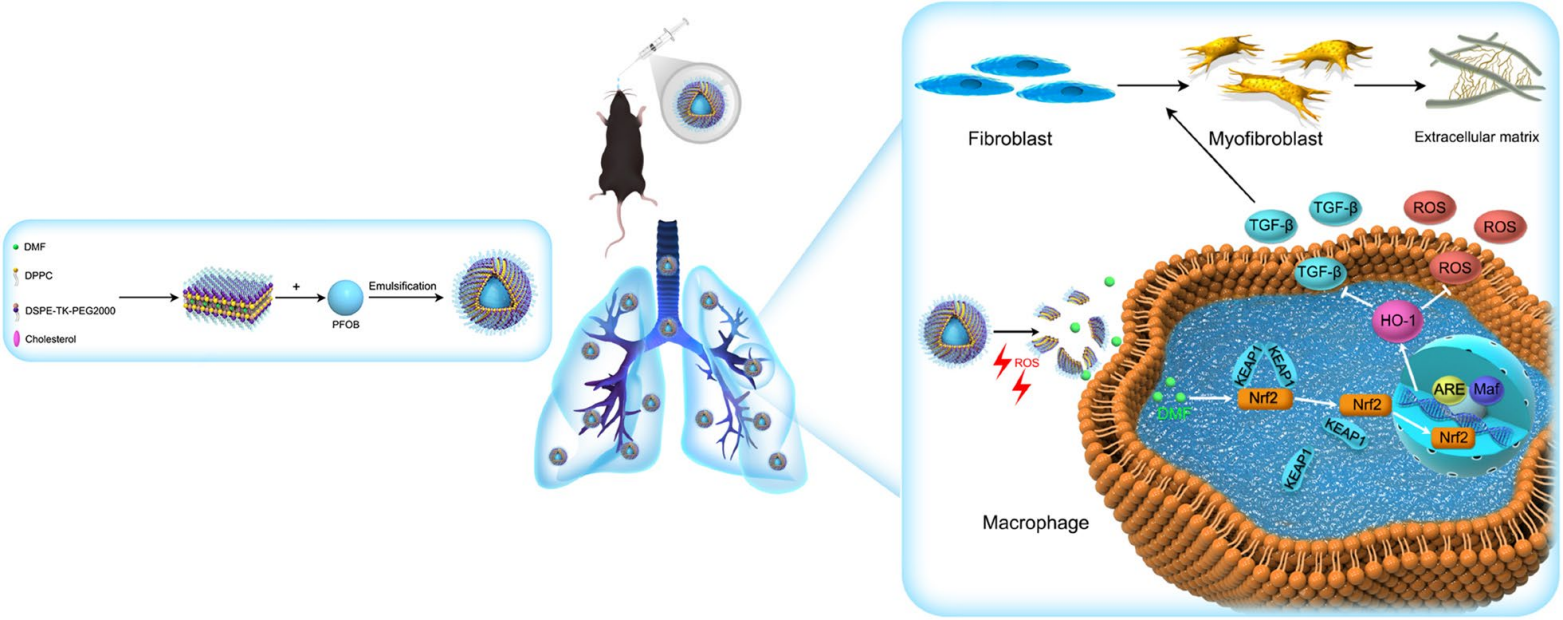

**Supplementary Information:**

The online version contains supplementary material available at 10.1186/s12951-022-01435-4.

## Introduction

Idiopathic pulmonary fibrosis (IPF) is an age-related, progressive interstitial lung disease with increasing incidence worldwide. As the global population ages, the economic burden of IPF is proposed to increase [1,2]. The prognosis of patients diagnosed with IPF is poor, with the median survival of no more than 5 years [3,4]. Nintedanib and pirfenidone are recommended for the treatment of IPF, but until now, none of them has shown any benefit for survival or improved life quality [2,5]. IPF is characterized by the irreversible destruction of the alveoli and bronchus and excessive extracellular matrix (ECM) deposition in the lung parenchyma. During aberrant remodeling and fibrosis, fibroblasts differentiate into myofibroblasts, and as a profibrotic phenotype, myofibroblasts are responsible for the synthesis of excessive ECM components, such as collagen and fibronectin [6–8]. Therefore, fibroblast-to-myofibroblast transition plays a key role in the pathophysiologic process of fibrosis development.

Innate and adaptive immune cells have been proven to widely participate in fibroblast-to-myofibroblast transition. As one of the most important types of innate immune cells in lung tissue, macrophages have been found to accumulate in the fibrotic area and are widely involved in fibrosis development [9–11]. In the early phase of fibrosis, macrophages mainly manifest the classically activated phenotype (M1), which generates various proinflammatory cytokines to exacerbate the recurrent microinjury of alveolar epithelial cells [12]. In the injury healing and fibrosis phase, macrophages undergo phenotypic transformation from the M1 type to the M2 type under the stimulation of Th2 cytokines such as interleukin (IL)-4 and IL-13 and produce a range of cytokines such as transforming growth factor-β (TGF-β), platelet derived growth factor (PDGF), chemokine ligand 17, and chemokine ligand 18 [13,14]. TGF-β is a typical "profibrotic" cytokine that induces fibroblast differentiation into myofibroblasts and promotes collagen production in lung tissue [9,15]. Moreover, reactive oxygen species (ROS) produced by macrophages are considered to be key factors in the pathogenesis of pulmonary fibrosis [16,17]. In addition, ROS can promote the profibrotic polarization of macrophages [18–20].

Nuclear factor erythroid 2 related factor 2 (Nrf2) is a key mediator of the system defense against oxidative stress [21]. Under physiological conditions, Nrf2 is anchored by Kelch-like ECH-associated protein 1 (Keap1) in the cytoplasm ^[[[22]]]^. However, in the case of oxidative stress, the Nrf2-Keap1 interaction dissociates, and activated Nrf2 enters the nucleus, forms a dimer with the Maf protein, and then interacts with antioxidant response elements (ARE) to activate heme oxygenase-1 (HO-1), thereby inhibiting ROS production [23,24]. In addition, the suppressive effect of Nrf2 on TGF-β expression has also been confirmed [25,26]. Some studies have demonstrated therapeutic effects on fibrotic diseases, including pulmonary fibrosis, through Nrf2 and HO-1 expression modulation [27–31]. As ROS and TGF-β are closely associated with fibrosis development, this evidence indicates that the modulation of Nrf2-HO-1 signaling might be a promising therapeutic strategy for IPF.

Dimethyl fumarate (DMF) is approved by the Food and Drug Administration (FDA) for the first-line treatment of relapsing–remitting multiple sclerosis [32]. Animal experiments have revealed that DMF has antioxidant functions through Nrf2 and HO-1 activation [21,27,33]. Although DMF has good biological safety, systemic application has the risk of potential systemic adverse reactions. Local drug administration can reduce the accumulation of drugs in other organs of the body and improve therapeutic efficacy. Inhalation for pulmonary drug delivery can provide drugs directly to lung lesions, reducing potential adverse reactions, and is currently receiving increasing attention. Some antifibrotic inhaled drugs were initially used in clinical trials, which further proves that inhalation therapy is very promising for drug delivery for pulmonary fibrosis [2]. But inhaled particles can be swept out of the lung along with mucus by cilia in the trachea and bronchi or phagocytosed by alveolar macrophages, which affects their therapeutic effect. Therefore, the designed inhalation-based drug delivery system should be able to overcome these physiological barriers and prolong the accumulation time of the drug in the lungs, thereby improving the therapeutic effect.

The application of nanotechnology has improved the effectiveness of pulmonary delivery of drugs. Liposomes have excellent safety and biocompatibility, studies have shown that inhaled drugs loaded in liposomes have a prolonged residence time in the lungs, thereby enhancing the therapeutic effects in lung tissues and minimizing systemic exposure [34–36]. Moreover, an inhaled liposome suspension has been approved by the FDA for clinical treatment, which strongly demonstrated the liposome as a promising nanoplatform for inhalation-based drug delivery [34]. Herein, we designed and synthesized a ROS-responsive liposome for DMF delivery in the fibrotic lung. Due to the high concentration of ROS in the lungs of pulmonary fibrosis, an ROS-responsive nanoplatform is a good choice for drug delivery. We linked DSPE and PEG_2000_ with the ROS-sensitive linker thioketal (TK) to form DSPE-TK-PEG_2000_. Using this material, we synthesized DSPE-TK-PEG_2000_@DMF liposomes (DTP@DMF NPs) with DMF encapsulated in the shell and perfluorooctyl bromide (PFOB) wrapped in the core of the NPs. The TK bond can be oxidized in a high-level ROS environment, which destroys the hydrophilic shell of liposomes and facilitates DMF release in the fibrotic area [37–39]. PFOB can increase the stability of liposomes with ideal biocompatibility [40]. After intratracheal instillation, these liposomes can attenuate lung fibrosis development by activating Nrf2-HO-1 signaling in macrophages to suppress TGF-β and ROS production.

## Methods

### Materials

Acetone, 3-mercaptopropionic acid, DSPE and PEG_2000_ were obtained from RuixiBio (Xi’an, China). DMF and PFOB were purchased from Sigma-Aldrich (St. Louis, USA). Antibodies against Nrf2, collagen Ia1, α-smooth muscle actin (SMA), F4/80, and CD206 were purchased from Cell Signaling Technology (MA, USA). CD86 was purchased from Invitrogen (Carlsbad, CA, USA). Antibodies against GAPDH and HO-1 were purchased from Proteintech (Hubei, China). The HR-conjugated goat anti-rabbit secondary antibody was obtained from Abbkine Scientific (Wuhan, China). Recombinant IL-4 was purchased from BioLegend (CA, USA). The ELISA kits for TGF-β, IL-4, and IL-13 were purchased from Multisciences (Lianke) Biotech (Hangzhou, China), and the test kits for superoxide dismutase (SOD) and malondialdehyde (MDA) were purchased from Beyotime Biotechnology Company (Shanghai, China). DiR was purchased from Bioss (Beijing, China), and DiI and 4',6-diamidino-2-phenylindole (DAPI) were purchased from Beyotime Biotechnology Company (Shanghai, China). Other reagents used in western blot analysis were purchased from Solarbio (Beijing, China).

### Synthesis of TK

A mixture of 3-mercaptopropionic acid (1.78 g, 16.8 mmol), acetone solution (2.0 g, 11.08 mmol) and catalytic tallow fatty acid was stirred at room temperature for 12 h under a nitrogen atmosphere. The mixture was placed on ice until complete crystallization to quench the reaction. The precipitate was filtrated and washed with hexane and cold water and dried to obtain the product.

### Synthesis of DSPE-TK-PEG_2000_

The synthesis procedure was modified based on previous reports [37,38]. TK (39.0 mg, 0.15 mmol) and 4-dimethylaminopyridine (DMAP) (22.6 mg, 0.185 mmol) were dissolved in 7 ml anhydrous dimethyl sulfoxide. Dicyclohexyl carbodiimide (DCC) (190.5 mg, 0.93 mmol) was dissolved in 3 ml anhydrous dimethyl sulfoxide and added to the TK solution slowly. The mixture was stirred at 60 °C for 60 min. Then, 115.5 mg DSPE_2000_ was dissolved in 3 ml anhydrous DMSO and added to the solution. The reaction continued at 60 °C for 24 h under a nitrogen atmosphere. Then, 309 mg methoxy polyethylene glycol (PEG_2000_) dissolved in 3 ml anhydrous DMSO was added and incubated for another 24 h. The resulting precipitate was further dissolved in 10 ml of DMF and dialyzed (MWCO 7 kDa, Spectrum Laboratories, Laguna Hills, CA) against deionized water. The final product was obtained after freeze-drying under vacuum for 12 h.

### Synthesis of DP@DMF and DTP@DMF NPs

The DP@DMF NPs, DTP@DMF NPs and DTP NPs were generated through a one-step emulsion method as previously reported [41,42]. Then, 12 mg of DPPC, 4 mg of DSPE-TK-PEG_2000_, 4 mg of cholesterol and 1 mg of DMF were dissolved in 16 ml of CHCl_3_. After transferring the CHCl_3_ solution to a round flask, the round flask was placed on a rotary evaporator (Yarong Inc, Shanghai, China), and rotary evaporation was continued for 2 h to form a lipid film on the bottom of the round flask. Then, 3 ml of double distilled water was added to the flask to hydrate the lipid membrane. After adding 200 μl of PFOB, the suspension was emulsified for 5 min in an ice-water bath (power: 100 W, 5 s on and 5 s off) using a sonicator (Sonics & Materials Inc., USA). Finally, the nano solution was centrifuged at 7,000 rpm for 5 min at 4 °C, and after three repetitions, DTP@DMF NPs were obtained. DSPE-TK-PEG_2000_ was replaced by DSPE-PEG_2000_ to prepare DP@DMF NPs, no DMF was added to prepare DTP NPs, and an additional 0.5 mg of DiR was added to CHCl_3_ before rotary evaporation to prepare DiR-labeled DTP@DMF NPs. The other synthesis steps were the same, and after the synthesis was completed, the NPs were stored at 4 °C in the dark.

### Characterization

Transmission electron microscopy (TEM, Hitachi-7500, Japan) was applied to observe the structure of the NPs. The particle size and zeta potential of DTP@DMF NPs were tested by a particle size and zeta potential analyzer instrument (NanoBrook Omni, Brookhaven Instrument Ltd, UK). Samples were equilibrated at 25 °C for 60 s before measurement. The chemical structures of DSPE-TK-PEG and DSPE-PEG were confirmed using a ^1^H nuclear magnetic resonance (NMR) spectrometer (NEO-600, Bruker Bios pin. Germany). To test the stability of DTP@DMF NPs, the NPs were freeze-dried for 24 h by a freeze-drier (FD-1A-80, Bilon Instrument, China) and stored at − 20 °C. The size distribution, zeta potential, drug encapsulation capacity, and loading capacity were analyzed and calculated after rehydration. To test the concentrations of DMF, DMF was dissolved in DMSO at different concentrations, and was tested by an ultraviolet–visible-near infrared (UV–Vis-NIR) spectrophotometer (UV-3600, Shimadzu, Japan). Standard curves of DMF were drawn based on the peak values UV–Vis-NIR spectra with different concentrations. To calculate the encapsulation efficiency (EE) and drug loading capacity (DLC), DTP@DMF NPs were separated by centrifugation at 8000 rpm for 5 min, and then, the precipitated DTP@DMF NPs were ruptured by 3 ml of DMSO. Finally, the mixture was centrifuged at 8000 rpm for 5 min, and the concentration of DMF in DMSO was measured by UV–vis-NIR spectrophotometer.

The EE of DMF in DTP@DMF NPs was calculated according to the formula:

EE (%) = (weight of DMF in DTP@DMF NPs/weight of DMF in feeding) × 100%

The DLC of DMF in DTP@DMF NPs was calculated by the formula:

DLC (%) = (weight of DMF in DTP@DMF NPs/weight of DTP@DMF NPs) × 100%.

### ROS-responsive drug release

The DTP@DMF NPs were dissolved in 6 ml of deionized water or deionized water with 1 μM, 10 μM, and 100 μM H_2_O_2_. After 0, 1, 3, 5, 7, and 9 h of the reaction, 1 ml of the solution was removed, and the EE of the NPs was measured to calculate the ROS-triggered drug release. The EE calculation was consistent with the method described above. After incubating with 100 μM H_2_O_2_ for 9 h, the size distribution of DTP@DMF NPs were reanalyzed, and the morphology of NPs was observed through TEM.

Another experiment was conducted in order to better reflect the drug release profile of DTP@DMF NPs in the presence and absence of H_2_O_2_. The NPs in the deionized water with the H_2_O_2_ concentration of 100 μM were centrifuged and transferred to water without H_2_O_2_ at 1, 3, and 5 h after the reaction. The reaction continued for 1 h, and then the DTP@DMF NPs were transferred back to the deionized water with H_2_O_2_ concentration of 100 μM. At 0, 1, 3, 5, and 7 h after the reaction, 1 ml of mixture was taken out for drug release test.

### Animal models

Male C57BL/6 mice (6–8 weeks old) were purchased from Chongqing Medical University (Chongqing, China). All mice were housed in a specific pathogen-free animal facility with a 12:12 h light/dark photocycle at Chongqing Medical University. Mice were fed rodent food and had free access to water. The animal experiments in this study were approved by the Animal Care and Use Committee of Chongqing Medical University and in accordance with the National Institutes of Health Guide for the Care and Use of Laboratory Animals.

Program 1: To study the association between macrophage polarization and the development of pulmonary fibrosis, the mice were divided into five groups (5 mice in each group): (1) the control group, (2) the 7-day group, (3) the 14-day group, (4) the 21-day group, and (5) the 28-day group. The mice received saline or bleomycin (BLM) intratracheal instillation (2.5 mg/kg, 50 μl) and were sacrificed after 7, 14, 21, or 28 days.

Program 2: To investigate the therapeutic effect of DTP@DMF NPs, mice were randomly divided into five groups: (1) the control group instilled with 50 μl PBS, (2) the BLM group instilled with BLM, (3) the low-dose DTP@DMF group, (4) the medium-dose DTP@DMF group, and (5) the high-dose DTP@DMF group. Fourteen days after BLM induction, the mice received 50 μl DTP@DMF with a DMF concentration of 0.5, 2, or 5 mg/kg. The drug instillation conducted every three days in the 14-day treatment period.

Program 3: To compare the therapeutic effect of DTP@DMF NPs at different phases of fibrosis, mice were randomly divided into six groups. Mice in the three groups received BLM instillation and were sacrificed after 28, 35, and 42 days. In the other three groups, mice received a 14-day treatment of DTP@DMF NPs intratracheal instillation every three days beginning on 14, 21, and 28 days after BLM treatment, respectively.

Program 4: To compare the therapeutic effects of inhalation and systemic administration of DTP@DMF NPs and DMF on pulmonary fibrosis, mice were divided into three groups: (1) the BLM group, (2) the inhalation administration group, and (3) the systemic administration group. Fourteen days after BLM instillation, mice received PBS 50 μl inhalation, DTP@DMF NPs (DMF 5 mg/kg) 50 μl inhalation, or DMF (50 mg/kg) 50 μl intravenous administration every three days, respectively. The administration continued for 14 days.

Program 5: To compare the therapeutic effect of DTP@DMF NPs with other NPs on pulmonary fibrosis in vivo, mice were randomly divided into six groups: (1) the control group, (2) the BLM group, (3) the DMF group, (4) the DP@DMF NPs group, (5) the DTP@DMF NPs group, and (6) the DTP NP group. The mice received 50 μl PBS, DMF and NPs (with a DMF concentration of 5 mg/kg except in the DTP NPs group) treatment 14 days after BLM instillation. The drug instillation conducted every three days in the treatment period.

At the designated timepoints in each program, the mice were euthanized. The left lung was harvested and then separated into two pieces. One piece was kept in 4% neutral paraformaldehyde for histopathological analysis, and the other piece was frozen at − 80 °C for western blot, SOD and MDA analysis. The left trachea was ligated, and bronchoalveolar lavage fluid (BALF) was performed on the right lung three times with 1 ml of saline; the lavage fluid was centrifuged at 1200 rpm for 5 min, and the supernatant was kept at − 20 °C for cytokine detection.

### In vivo biodistribution of the liposomes

Mice with pulmonary fibrosis were treated with the DiR-loaded DTP@DMF NPs (DMF concentration: 5 mg/kg) via intratracheal instillation. The mice were imaged at certain time points (10 min, 30 min, 1 h, 2 h, 4 h, 6 h, 8 h, 9 h) by the Maestro in vivo imaging system (Cambridge Research &Instrumentation, Inc., Woburn, MA, USA). After 9 h, the mice were sacrificed, and the major organs were collected for ex vivo fluorescence imaging.

### Cell culture and treatment

The mouse macrophage RAW264.7 cell line and mouse fibroblast NIH-3T3 cell lines were obtained from Chongqing Key Laboratory of Ultrasound Molecular Imaging, Chongqing Medical University. The RAW264.7 and NIH-3T3 cell lines were cultured in DMEM with 10% FBS. Cells were grown at 37 °C in a humidified 95% air/5% CO_2_ incubator.

RAW264.7 cells were plated into 6-well plates (3 × 10^5^ per well), pretreated with IL-4 (25 ng/ml) for 48 h, and then exposed to DTP@DMF NPs with DMF concentrations of 15, 30, 60, and 120 µg/ml for 24 h. These concentrations were approved for the biosafety of RAW264.7 cells by the Cell Counting Kit-8 (CCK-8) assay. After treatment, the culture medium was collected to detect the IL-4, IL-13, TGF-β, SOD, and MDA levels, and RAW264.7 cells were harvested for western blot analysis. We then performed a coculture experiment to explore the effect of macrophage RAW264.7 cells on fibroblast NIH-3T3 cells. This experiment was conducted using the Transwell® (24 mm, pore size: 0.4 μm, Corning Incorporated, USA). Briefly, DTP@DMF NPs at various DMF concentrations (15, 30, 60, and 120 µg/ml) were added to IL-4-pretreated RAW264.7 cells for 24 h and cocultured with NIH-3T3 cells for 24 h. After coculture, the culture medium and cells were collected for ELISA and western blot analysis.

To compare the difference of therapeutic effect between DMF, DP@DMF NPs, DTP@DMF NPs, and DTP NPs, various NPs with DMF concentration of 120 µg/ml were added into IL-4-pretreated RAW264.7 cells. The detailed experimental procedures were the same as above.

### Cellular endocytosis comparison between DP@DMF NPs and DTP@DMF NPs

For fluorescence microscope observation, the RAW264.7 cells were incubated with DiI-labeled DP@DMF NPs and DTP@DMF NPs with DMF concentration of 120 µg/ml. After 0, 2 and 4 h of incubation, RAW264.7 cells were labeled with DAPI for 10 min, and then washed 3 times. The cellular endocytosis of DP@DMF NPs and DTP@DMF NPs by RAW264.7 cells were observed through fluorescence microscope.

For flow cytometry analysis, RAW264.7 cells were incubated with DiI-labeled DP@DMF NPs and DTP@DMF NPs (DMF: 120 µg/ml). After 0, 2 and 4 h, cells were harvested and resuspended in the test tube for analysis.

### In vivo toxicity studies of DTP@DMF NPs

Mice with no BLM conduction were randomly divided into four groups and received DTP@DMF NPs (DMF concentration 5 mg/kg) intratracheal instillation. Mice were received a period of 14-day treatment in our study, so we tested the potential effect of DTP@DMF NPs on mice from the start of treatment to one week after the end of treatment. Therefore, the mice were instilled with DTP@DMF NPs every three days, and after 0, 7, 14, and 21 days, mice were sacrificed, blood samples and major organs were collected for blood and histopathological analysis.

### Histopathological analysis

Lung tissue was placed in 4% neutral paraformaldehyde, fixed at room temperature for 24 h, and then embedded in paraffin. Lung tissue was cut into 4 µm sections and stained with hematoxylin and eosin (H&E) staining and Masson’s trichrome staining as previously reported. The severity of pulmonary fibrosis was assessed using the Ashcroft score [43]. For immunohistochemistry, the sections were treated with anti-F4/80, anti-CD86, and anti-CD206 antibodies. Then, the slides were placed overnight at 4 °C, washed three times, and incubated with HRP-conjugated secondary antibody.

For immunofluorescence, the sections were incubated with anti-Nrf2 and anti-HO-1 antibodies, and the corresponding anti-mouse and anti-rabbit antibodies were chosen as the secondary antibodies. Finally, the slides were incubated with DAPI for 3 min. Then, the slides were analyzed under a fluorescence microscope.

### Western blot analysis

Protein was extracted from lung tissue and cells in RIPA lysis buffer (Beyotime) containing 100 × protease inhibitor cocktail (Beyotime) as previously reported. Proteins were loaded with equal amounts (30 µg) and separated by 10% or 7.5% polyacrylamide gel electrophoresis according to the molecular weight of the protein. After that, proteins were transferred to a PVDF membrane and blocked with 5% nonfat milk for 2 h. The PVDF membrane was washed 3 times and then incubated with the primary antibodies overnight at 4 °C. The membranes were washed three times and then incubated with the corresponding goat anti-rabbit secondary antibody for 1 h at room temperature. Finally, the membranes were washed 3 times, and the protein signals were revealed with an enhanced chemiluminescence system (Thermo Scientific).

### Cytokine analysis

IL-4, IL-13 and TGF-β levels in BALF and SOD and MDA levels in lung tissue were measured by the assay kits according to the instructions.

### In Vitro Toxicity Assessments

The cell toxicity of DTP@DMF NPs was measured by CCK-8 assay. RAW264.7 cells and NIH-3T3 cells were seeded in 96-well plates (2000 cells/well) and cultured for 24 h. Then, 100 µl of DTP@DMF NPs at different DMF concentrations (15, 30, 60, and 120 µg/ml) was added to the plate. After incubating for 36 h, 10 μl of CCK-8 solution was added to each well to evaluate the cell viability using a microplate reader.

### Statistics

All statistical analyses were performed with SPSS (Version 24). One-way ANOVA was used for comparisons between groups. Data are expressed as the mean ± standard deviation, and *P* < 0.05 was considered to indicate statistical significance.

## Results and discussion

### Design, synthesis and characterization of NPs

**Fig. 1:**
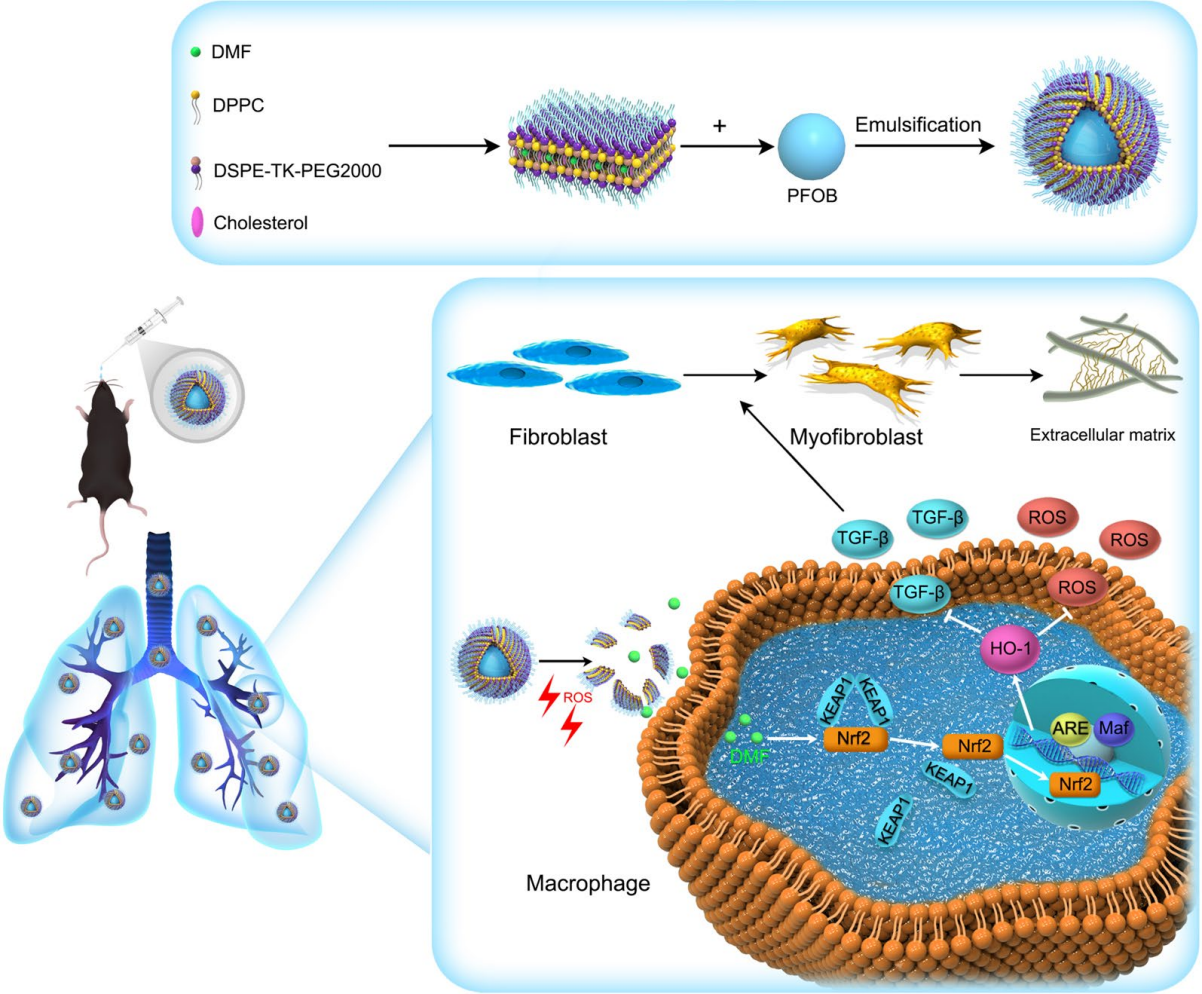
Schematic illustration of synthesis and the mechanism of the therapeutic effect of DTP@DMF NPs

**Fig. 2:**
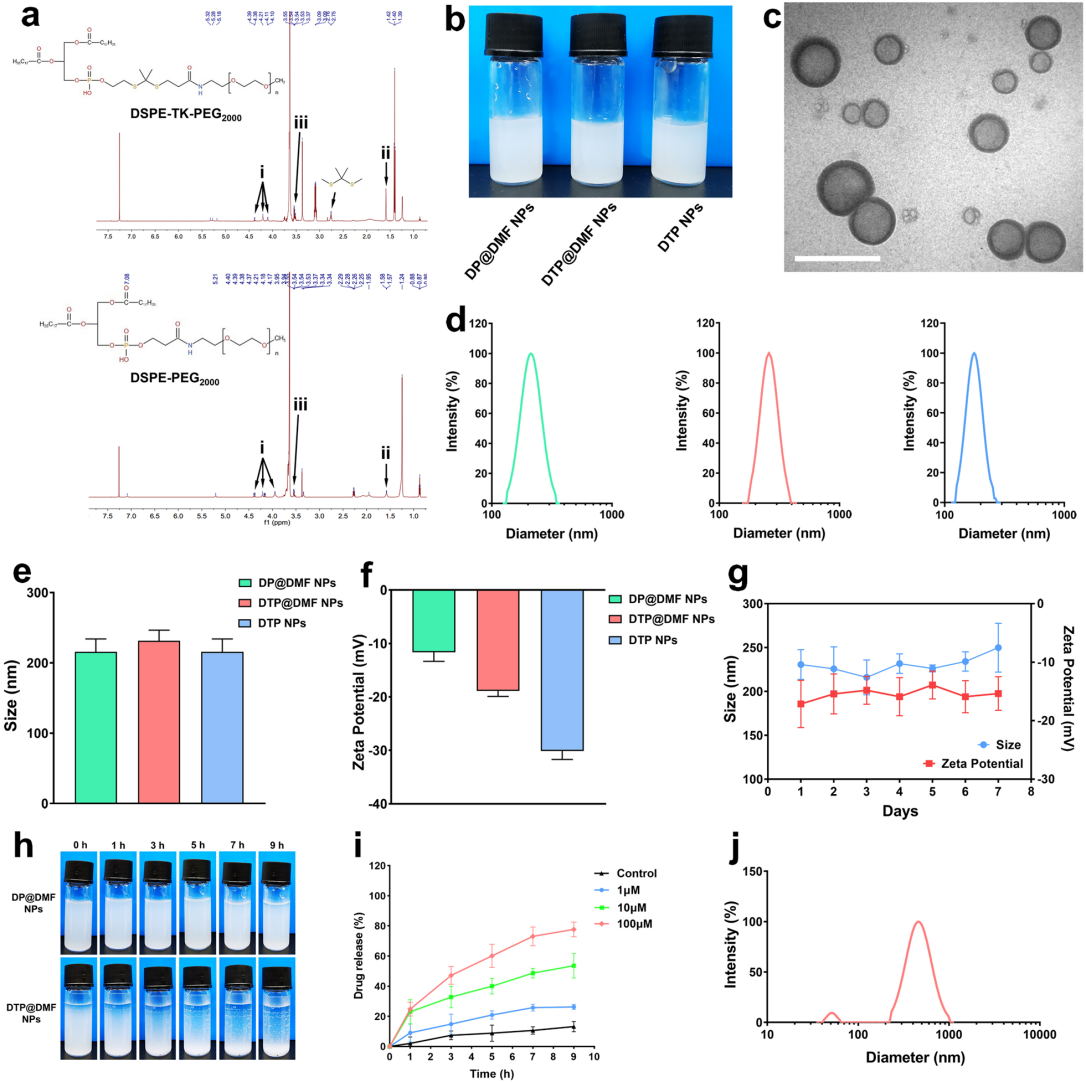
Synthesis and characterization of NPs. **a** The ^1^H NMR spectra of DSPE-TK-PEG_2000_ and DSPE-PEG_2000_. **b** The appearance of DP@DMF NPs, DTP@DMF NPs, and DTP NPs solution. **c** Representative TEM images of DTP@DMF NPs (scale bar: 500 nm). **d** Size distribution of DP@DMF NPs (green), DTP@DMF NPs (red), and DTP NPs (blue). **e** The average size and **f** zeta potential of NPs (n = 3). **g** The change of size and zeta potential of DTP@DMF NPs within 7 days (n = 3). **h** Changes in the appearances of DTP@DMF NPs and DP@DMF NPs in the presence of 100 μM H_2_O_2_. **i** The cumulative drug release curve of DTP@DMF NPs in the presence of 1 μM, 10 μM, and 100 μM H_2_O_2_. Data are expressed as the mean ± SD (n = 3). **j** Size distribution of DTP@DMF NPs after incubating with 100 μM H_2_O_2_ for 9 h

^1^H NMR was used to verify the structures of DSPE-TK-PEG and DSPE-PEG. The results of nuclear magnetic characterization are shown in Fig. 2a. The characteristic peak of the TK bond was observed at ~ 2.8 ppm, which was consistent with other reports [44]. The characteristic peak of PEG appeared at ~ 4 ppm (black arrow i), and the peaks of DSPE were observed at ~ 1.5 and ~ 3 ppm (black arrow ii, iii). These data confirmed the successful synthesis of DSPE-TK-PEG. The DP@DMF NPs, DTP@DMF NPs and DTP NPs were fabricated using DSPE-TK-PEG and DSPE-PEG as the main materials. The synthesis illustration is shown in Fig. 1. Because of the lipophilic property, DMF was integrated into the lipid bilayer shell of NPs during the synthesis procedure, which may protect DMF from clearance mediated by the monocyte-macrophage system or by the mucus in the trachea to achieve higher drug concentrations at the site of pulmonary fibrotic lesions [34]. PFOB was encapsulated in the shell of NPs as the core to increase the stability. Finally, the characteristics of the NPs were assessed.

The DP@DMF NPs, DTP@DMF NPs and DTP NPs solutions appeared milky white (Fig. 2b). The DTP@DMF NPs had a regular spherical morphology and core/shell structure with uniform size, as revealed by TEM (Fig. 2c). The size and zeta potential analyses revealed that the mean diameters of DP@DMF NPs, DTP@DMF NPs and DTP NPs were 215.60 ± 18.41 nm, 231.50 ± 14.93 nm and 195.19 ± 7.18 nm, respectively (Fig. 2d, e). The size of NPs increased slightly after encapsulation with DMF, while no obvious difference was found for DP@DMF NPs and DTP@DMF NPs. The average zeta potential values of the above three NPs were − 11.65 ± 1.70 mV, − 17.72 ± 1.58 mV and − 30.24 ± 1.60 mV, respectively (Fig. 2f). The appropriate size and negative potential prevent NPs from aggregating and facilitate NPs to effectively stay longer in tissue [45]. Furthermore, we assessed the average size and zeta potential change of NPs over 7 consecutive days. As shown in Fig. 2g, no obvious change in size or zeta potential was found within 7 days. In addition, no obvious change in the appearance of liposome solutions was observed (Additional file 1: Fig. S1).

After the freeze-drying and rehydration, the mean diameter of DP@DMF NPs was 292.66 ± 13.51 nm, and the zeta potential was − 18.80 ± 5.81 mV. The freeze-drying method increased the stability as well as the extended storage time of DTP@DMF NPs. In addition, the NPs can be easily rehydrated before use. This improves the prospects of DSPE-TK-PEG@DMF NPs for industrial production, storage, and clinical application. The UV–Vis-NIR absorption spectra of DMF and liposomes were shown in Additional file 2: Fig. S2, and the UV–Vis-NIR absorption spectra at different concentrations are shown in Additional file 3: Fig. S3. After calculation, the encapsulation efficiency and drug loading efficiency of DTP@DMF NPs were 75.85% and 3.61%, respectively. For the DSPE-TK-PEG@DMF NPs experiencing freeze-drying and rehydration, the encapsulation efficiency and drug loading efficiency was 66.79% and 3.18%.

ROS-responsive drug release is a characteristic feature of DTP@DMF NPs, so we examined the drug release property of DTP@DMF NPs under ROS stimulation. The NPs were mixed with H_2_O_2_ solution at different concentrations (1 μM, 10 μM and 100 μM) to simulate the H_2_O_2_ atmosphere. The encapsulation efficiency of DMF was tested at different timepoints to evaluate drug release. After 9 h of incubation with 100 μM H_2_O_2_ solution, the DTP@DMF NPs emulsion turned into a transparent solution, while no obvious change was found for the milky white appearance of DP@DMF NPs (Fig. 2h). Figure 2i demonstrates the drug release characteristics of DTP@DMF NPs in H_2_O_2_ solution. Sustained and slow drug release was observed without H_2_O_2_, while increased drug release behavior was observed under a H_2_O_2_ atmosphere, and the release speed was dependent on the H_2_O_2_ concentration. At a concentration of 1 μM, approximately 26.2% of DMF was released from DTP@DMF NPs after 9 h. When the concentration was raised to 10 μM and 100 μM, the NPs showed significant drug release, with drug release rates of approximately 53.6% and 77.7% within 9 h, respectively.

Moreover, we measured the size distribution of DTP@DMF NPs after incubating with 100 μM H_2_O_2_ for 9 h. The homogeneity of the particle size of DTP@DMF NPs was disappeared (Fig. 2j). The TEM images showed that the DTP@DMF NPs were almost completely broken, with debris scattered in the visual field and some were aggregated together (Additional file 4: Fig. S4). In order to better reflect the drug release profile of DTP@DMF NPs, we tested the DMF release in the presence and absence of H_2_O_2_. As shown in Additional file 5: Fig. S5, a remarkable decrease of the DMF release speed was observed after transferring DTP@DMF NPs to the deionized water without H_2_O_2_ at 1, 3, and 5 h. In our study, the concentration of H_2_O_2_ causing the drug release was similar to that in other studies [37,38]. Under the ROS stimulation, the TK bond were oxidized and broken into -SH, which damaged the hydrophilic shell layer and leading to the breaking of the NPs [38]. For DTP@DMF NPs, the DMF was encapsuled in the two hydrophilic shell layers. After the cleavage of TK bond, the integrity and stability of hydrophilic shell layer was disrupted, the subsequently breaking of DTP@DMF NPs caused the release of the DMF. These data demonstrated that the synthesized NPs have an ideal size, zeta potential and colloidal stability in electrolyte solutions. Moreover, the DTP@DMF NPs presented excellent ROS-responsive drug release properties under ROS stimulation.

### Macrophage accumulation and polarization are associated with pulmonary fibrosis development

**Fig. 3:**
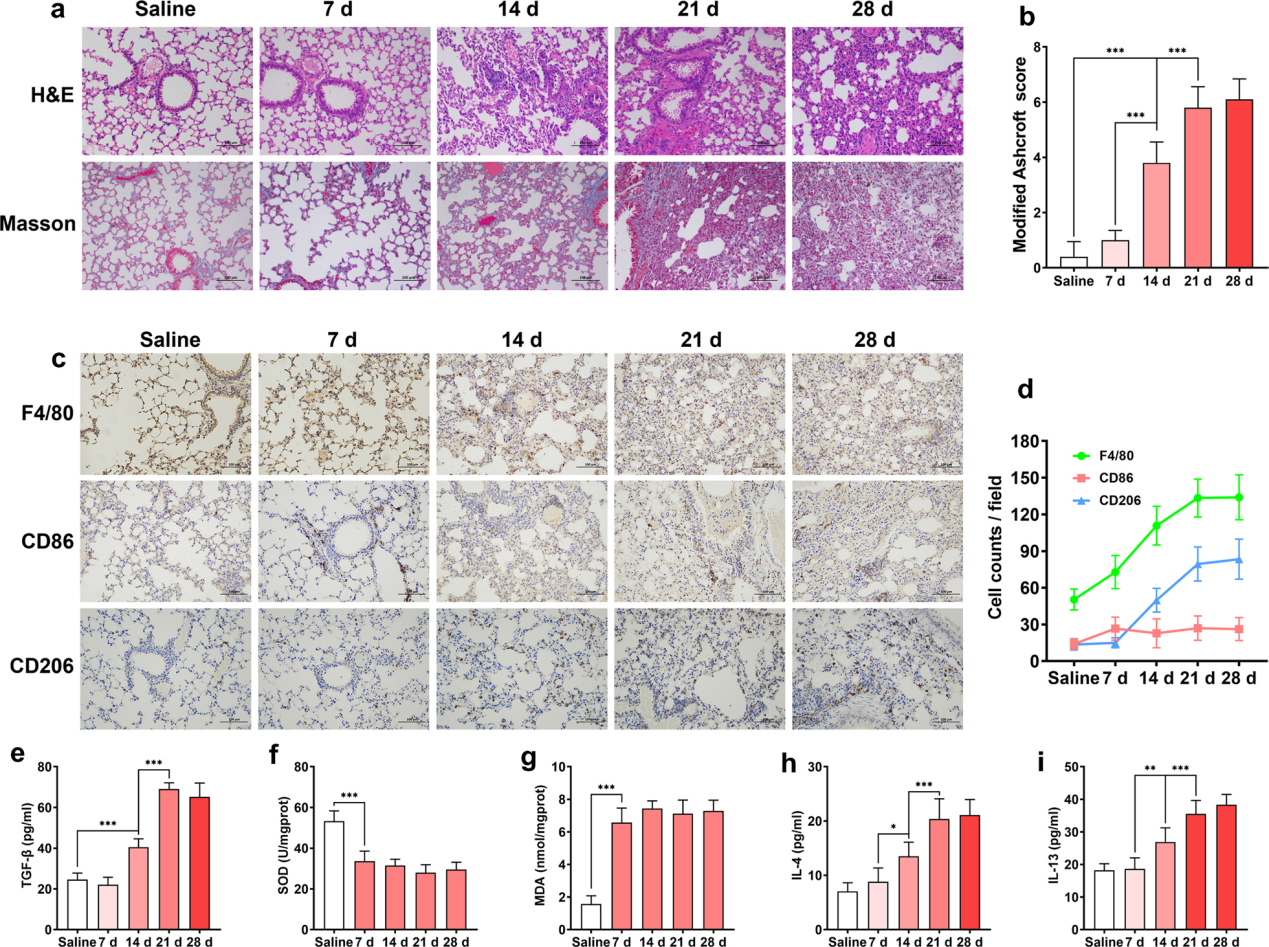
Dynamic changes in macrophage accumulation and polarization with the progression of pulmonary fibrosis. **a** Representative H&E and Masson’s trichrome staining of pulmonary fibrosis specimens at 7, 14, 21 and 28 days after BLM intratracheal instillation. **b** The severity evaluation score of fibrosis (n = 5). **c** Immunohistochemistry of F4/80^+^, CD86^+^, and CD206^+^ macrophages in pulmonary tissues and **d** quantification of macrophages and M1, M2 phenotypes during the fibrosis progression (n = 5). **e** Dynamic change of TGF-β, **f** SOD, **g** MDA, **h** IL-4, and **i** IL-13 levels as the lung fibrosis development (n = 5). Statistical analyses were performed using one-way ANOVA with S–N-K post hoc analysis. * *P* < 0.05, ** *P* < 0.01, *** *P* < 0.001

We first investigated the association between dynamic macrophage accumulation as well as polarization and pulmonary fibrosis development in a BLM-induced pulmonary fibrosis mouse model. After intratracheal instillation of BLM, no obvious pulmonary fibrosis was observed in the H&E staining and Masson’s trichrome staining of lung tissue on day 7. Fibrosis was first observed on day 14 after BLM administration, while gradually apparent fibrosis development was found on days 21 and 28. Masson’s trichrome staining images showed collagen deposition on day 14, which was consistent with the findings of H&E staining (Fig. 3a). Moreover, the severity of pulmonary fibrosis was quantitatively reflected by scoring and statistical analysis of H&E-stained pictures at each time point after BLM administration. We found that 14, 21, and 28 days after BLM administration, pulmonary fibrosis scores markedly increased (Fig. 3b). However, body weight loss of mice was found to occur on day 7 (Additional file 6: Fig. S6).

The accumulation and polarization of macrophages play an important role in the occurrence and development of fibrosis. Therefore, we assessed the total macrophages, M1-type macrophages, and M2-type macrophages based on the F4/80, CD86 and CD206 markers. Next, the dynamic changes in cell number during fibrosis development were analyzed. Moreover, the ratios of CD86^+^ macrophages (M1 type) and CD206^+^ macrophages (M2 type) were calculated. We found a slight increase in the number and proportion of M1-type macrophages on day 7 after BLM administration, which then declined over time. In contrast, the number and proportion of M2 macrophages increased significantly until 14 days. In addition, the total number of F4/80^+^ cells was increased in fibrotic lesions (Fig. 3c, d). TGF-β is the key factor of fibroblast-to-myofibroblast transition during fibrosis development. Although TGF-β can be produced by macrophages, fibroblasts, and other cell types, M2-type macrophages are the major source of TGF-β in the lung. As shown in Fig. 3e, a marked increase in TGF-β in BALF was found on days 14, 21 and 28. Oxidative stress caused by an imbalance of oxidants and antioxidants has been seen as the pathophysiological feature of lung fibrosis. As the major product of the oxidative stress, ROS may lead to the excessive consumption of antioxidants, and the over production of pro-oxidants [46]. The superoxide dismutase (SOD) is an important antioxidant, while malondialdehyde (MDA), the products of lipid peroxidation, was one of the major pro-oxidants in the body. Therefore, the change of SOD and MDA levels can well reflect the changes of oxidative stress and ROS production [46]. We found that SOD decreased remarkedly, while the MDA in the fibrotic area were maintained at high level during fibrosis development, indicating there is a microenvironment with high ROS concentration in fibrotic lung tissue (Fig. 3f, g). In addition, the levels of IL-4 and IL-13 in BALF increased in parallel with fibrosis severity (Fig. 3h, i). The above results suggested that macrophage accumulation and M2 polarization are closely related to the generation and development of pulmonary fibrosis, and the areas of pulmonary fibrosis support rapid drug release from ROS-responsive liposomes to achieve therapeutic effects.

### DTP@DMF NPs exert a therapeutic effect on pulmonary fibrosis

**Fig. 4:**
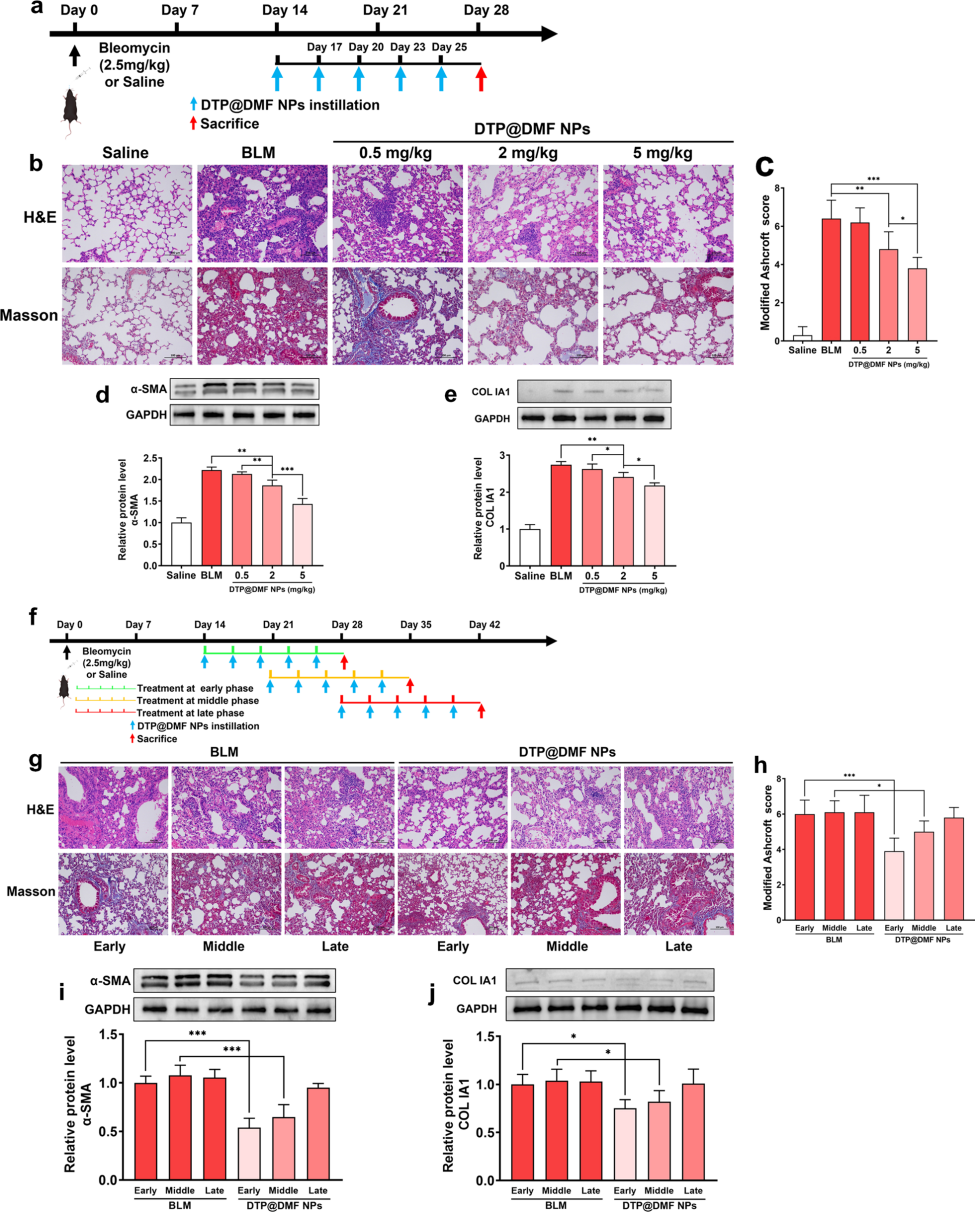
DTP@DMF NPs alleviate the pulmonary fibrosis development. **a** Schematic representation of the DTP@DMF NPs treatment design. **b** Representative H&E and Masson’s trichrome staining and **c** the severity evaluation score of pulmonary fibrosis after DTP@DMF NPs treatment (n = 5). Western blot analyses of **d** α-SMA and **e** collagen Ia1 levels in pulmonary fibrosis lesions (n = 3). **f** The schematic representation of the experimental design of DTP@DMF NPs treatment at different phases of fibrosis development. **g** H&E and Masson’s trichrome staining (scale bar: 100 μm) and **h** the severity score of fibrosis after intratracheal instillation of DTP@DMF NPs in different phases of fibrosis (n = 5). **i** α-SMA and **j** collagen Ia1 protein levels in fibrotic tissue (n = 3). Statistical analyses were performed via one-way ANOVA with S–N-K post hoc analysis. * *P* < 0.05, ** *P* < 0.01, *** *P* < 0.001

To evaluate the therapeutic effect of DTP@DMF NPs on BLM-induced lung fibrosis in mice, we treated mice with DTP@DMF NPs (0.5, 2, 5 mg/kg) on day 14 after BLM stimulation (Fig. 4a). Both the H&E and Masson’s trichrome staining images demonstrated that BLM-induced lung fibrosis can be attenuated by DTP@DMF NPs at doses of 2 mg/kg and 5 mg/kg (Fig. 4b, c). Moreover, western blot analysis also indicated that the levels of α-SMA and collagen I a1 were decreased in mice receiving intratracheal instillation of DTP@DMF NPs (Fig. 4d, e). In addition, DTP@DMF NPs instillation reduced the IL-4 and IL-13 level in BALF (Additional file 7: Fig. S7) and attenuated fibrosis caused body weight loss of mice in a dose-dependent manner (Additional file 8: Fig. S8). These results suggested that the DTP@DMF NPs can suppress myofibroblast differentiation and collagen production to attenuate fibrosis in a dose-dependent manner.

After validating the therapeutic effect of DTP@DMF NPs on fibrosis, we further compared the therapeutic effect on fibrosis at different phases during fibrosis progression. Consistent with findings from other studies [47], the early phase of fibrosis progression was observed on day 14, the fibrosis development phase and late phase of fibrosis development were approximately 14–21 days and 28 days, respectively. Therefore, mice received 14 days of DTP@DMF NPs (5 mg/kg) treatment on day 14, 21, and 28 after BLM instillation (Fig. 4f). The H&E and Masson’s trichrome staining images showed that the DTP@DMF NPs exerted a therapeutic effect on fibrosis development in the early and middle phases, especially in the early phase, the progression of pulmonary fibrosis was most efficiently blocked by DTP@DMF NPs (Fig. 4g, h). Accordingly, the collagen Ia1 and α-SMA expression levels were decreased in mice treated in the early and middle phases, while there was no significant decrease in mice treated with DTP@DMF NPs compared with mice treated with vehicle in the late phase (Fig. 4i, j). Besides, the DTP@DMF NPs treatment at early phase was associated with lower IL-4 and IL-13 level in BALF as well (Additional file 9: Fig. S9). Taken together, these results suggested that DTP@DMF NPs can suppress fibroblast-to-myofibroblast transition and reduce collagen deposition in lung tissue. Moreover, the optimal treatment period for DTP@DMF NPs is in the early and middle phases of pulmonary fibrosis.

### The DTP@DMF NPs decreased macrophage accumulation in fibrotic lung

**Fig. 5:**
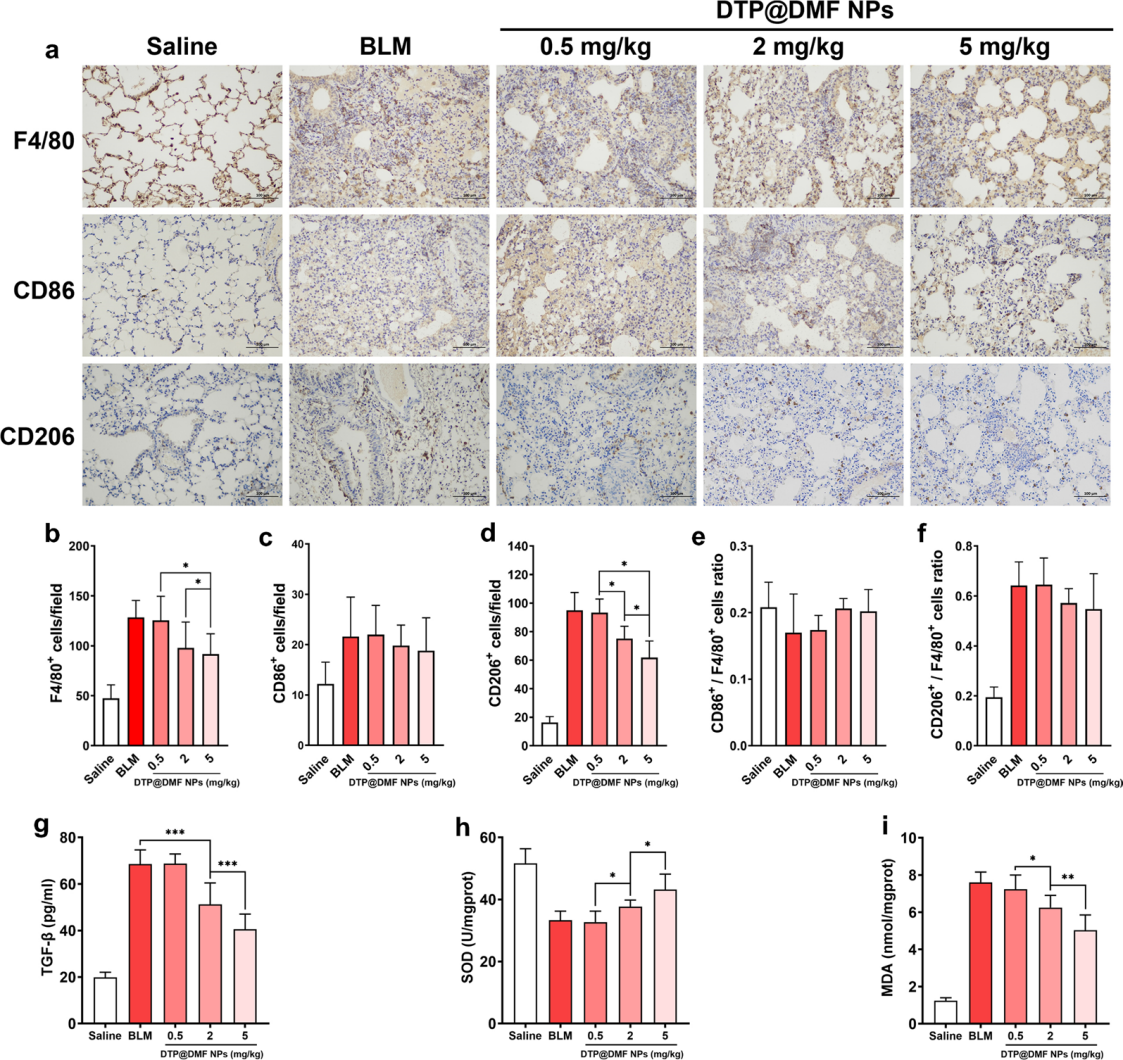
The effect of DTP@DMF NPs on the accumulation of activated macrophages. **a** Immunohistochemistry of F4/80^+^, CD86^+^, and CD206^+^ macrophages in lung tissues and **b**–**f** quantification of total macrophages and M1 and M2 phenotypes (n = 5). **g** TGF-β level in BALF, **h** SOD and **i** MDA levels in lung tissue (n = 5). Statistical analyses were performed using one-way ANOVA with S–N-K post hoc analysis. * *P* < 0.05, ** *P* < 0.01, *** *P* < 0.001

M2 macrophages can be recruited to the fibrotic area and activate fibroblast-to-myofibroblast transition through TGF-β and PDGF secretion [48]. In our study, we also demonstrated that the accumulation of M2 macrophages at the site of fibrosis is closely linked to the progression of pulmonary fibrosis. Therefore, we next investigated whether the DTP@DMF NPs can attenuated the accumulation of macrophages in fibrosis area. Mice received DTP@DMF NPs treatment on day 14 after BLM administration. After 28 days, the number of macrophages in fibrotic lungs was calculated. Treatment with DTP@DMF NPs significantly reduced the numbers of F480^+^ and CD206^+^ macrophages in the fibrotic area, while no effect on the number of CD86^+^ macrophages was observed (Fig. 5a–d). Regrettably, although there was a slight decrease in the CD206^+^ macrophage ratio, no statistically significant reduction in the CD206^+^ macrophage ratio or the CD86^+^ macrophage ratio was found (Fig. 5e, f). In addition, marked decrease of TGF-β in BALF, decrease of MDA and the increase of SOD level in lung tissue were found after DTP@DMF NPs treatment (Fig. 5g–i). Our results suggested that the DTP@DMF NPs inhibited the accumulation of M2-type macrophages, and suppressed M2 macrophage-related cytokine secretion in the fibrotic lung. Furthermore, the changes of SOD and MDA level indicated that the DTP@DMF NPs treatment could alleviate the ROS production in lung tissue.

### DTP@DMF NPs suppress macrophage-mediated fibroblast-to-myofibroblast transition by activating Nrf2-HO-1 signaling in macrophages

**Fig. 6:**
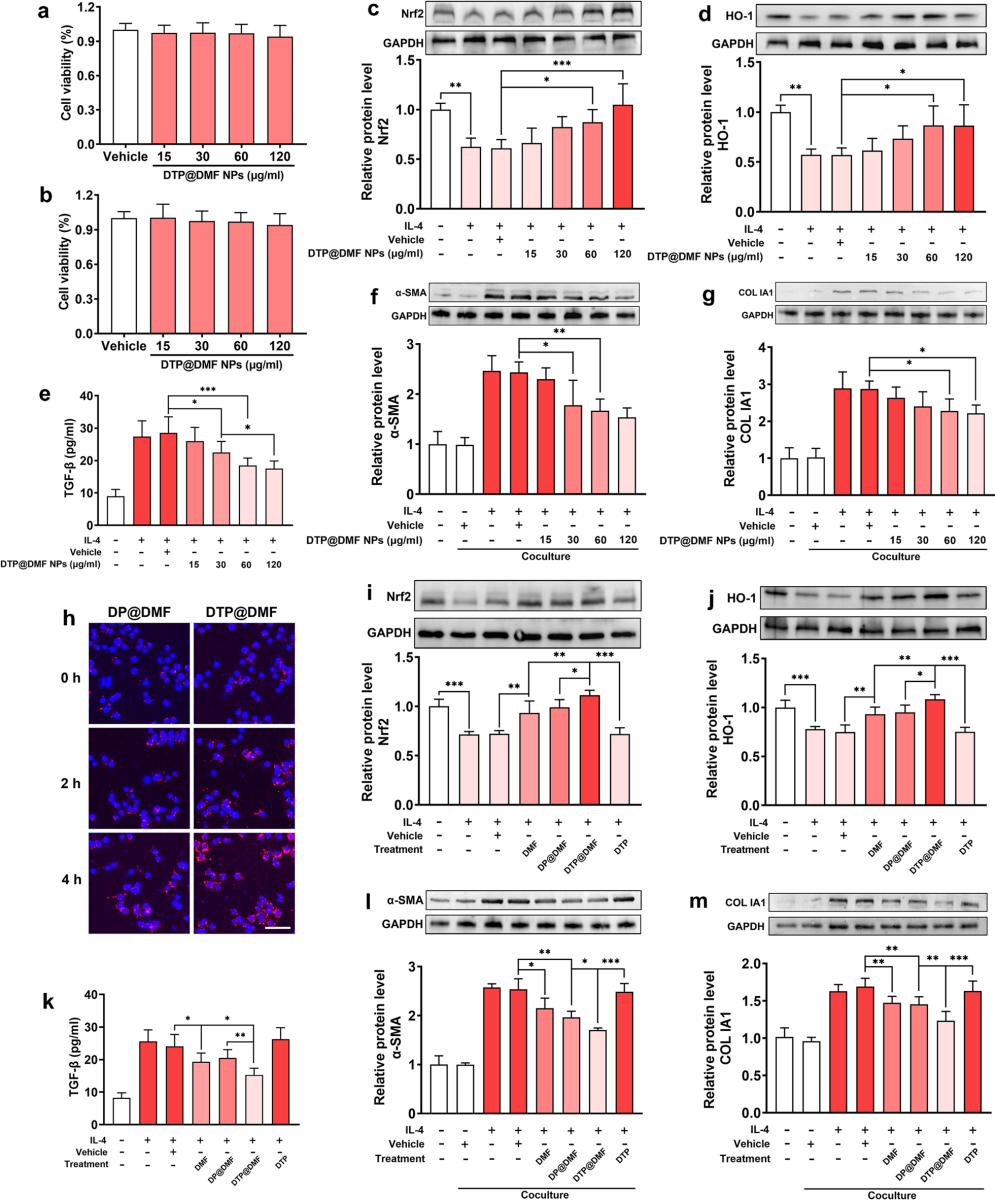
Macrophages attenuate myofibroblast transformation and collagen production via activating Nrf2-HO-1 signaling. The cytotoxicity of DTP@DMF NPs to **a** RAW264.7 cells, and to **b** NIH-3T3 cells. **c** Nrf2 and **d** HO-1 protein levels in RAW264.7 cells. **e** TGF-β concentration in RAW264.7 macrophage culture medium. **f** α-SMA and **g** collagen Ia1 protein levels in NIH-3T3 cells. **h** Cellular endocytosis of DP@DMF NPs and DTP@DMF NPs by RAW264.7 cells, scale bar = 20 μm. **i** The Nrf2 and **j** HO-1 protein levels in RAW264.7 cells treated with DMF, DP@DMF NPs and DTP@DMF NPs. **k** The TGF-β level in the culture medium of RAW264.7 cells. **l** The α-SMA and **m** collagen Ia1 protein levels in NIH-3T3 cells after DMF, DP@DMF NPs and DTP@DMF NPs administration. n = 3 for all the analysis. Statistical analyses were performed via one-way ANOVA with S–N-K post hoc analysis. * *P* < 0.05, ** *P* < 0.01, *** *P* < 0.001

We further identified the underlying mechanism by which DTP@DMF NPs delay the progression of pulmonary fibrosis. First, the CCK-8 assay showed no cytotoxicity of DTP@DMF NPs to both RAW264.7 and NIH-3T3 cells (Fig. 6a, b). Next, RAW264.7 cells were stimulated with IL-4 (25 ng/ml) for 48 h to induce M2 phenotype differentiation and received DTP@DMF NPs with the DMF concentrations of 15, 30, 60, and 120 µg/ml for another 24 h. Western blot analysis showed a marked decrease in Nrf2 and HO-1 expression after IL-4 stimulation, while the DTP@DMF NPs promoted Nrf2 and HO-1 expression in macrophage (Fig. 6c, d). Further analysis revealed that the TGF-β level in RAW264.7 medium supernatant was increased after IL-4 interference. In contrast, TGF-β expression was decreased in a dose-dependent manner by DTP@DMF NP treatment (Fig. 6e).

Next, we analyzed the effect of DTP@DMF NP treatment in a RAW264.7/NIH-3T3 cells coculture system. We found that macrophages with no IL-4 stimulation did not induce fibroblast-to-myofibroblast transition or collagen production in NIH-3T3 cells. However, after IL-4 stimulation, obvious α-SMA and collagen Ia1 expression was detected in NIH-3T3 cells by western blot analysis (Fig. 6f, g). The results of western blot analysis revealed that the transformation from fibroblasts to myofibroblasts was significantly inhibited by DTP@DMF NPs, and the secretion of collagen was also significantly reduced. In addition, we compared the endocytosis efficiency between DP@DMF NPs and DTP@DMF NPs. After incubating with IL-4 for 48 h, RAW264.7 cells were incubated with DP@DMF NPs or DTP@DMF NPs. The fluorescent images demonstrated the better endocytosis efficiency of DTP@DMF NPs than DP@DMF NPs after 2 h and 4 h incubation (Fig. 6h). The flow cytometry analyses demonstrated the consistent result as well (Additional file 10: Fig. S10). The PEG on the surface of NPs prevented the cellular uptake of NPs [49], while the cleavage of TK in the DTP@DMF NPs lead to the PEG attached to TK fell off from NPs, which might facilitate the endocytosis of NPs by RAW264.7 cells.

Further, we continued to compare the differences in the effects of direct DMF treatment and treatment with liposomes on RAW264.7 cells and NIH-3T3 cells in the coculture system. We found that the levels of Nrf2 and HO-1 in RAW264.7 cells treated with DP@DMF NPs were similar to those treated with direct administration of DMF, but the enhancement of Nrf2 and HO-1 by DTP@DMF NPs was better than that of DP@DMF NPs (Fig. 6i, j). The DTP@DMF NPs also had better inhibitory effects on TGF-β, SOD and MDA than DP@DMF NPs (Fig. 6k, Additional file 11: Fig. S11). Our findings are consistent with other reports that elevated Nrf2 and HO-1 expression could exert antioxidative stress effects [50]. Furthermore, the administration of DTP@DMF NPs significantly decreased the α-SMA and collagen-Ia1 expression in NIH-3T3 cells, while the DP@DMF NPs presented relatively poor efficacy on α-SMA and collagen-Ia1 decrease compared with DTP@DMF NPs. In addition, DTP NPs with no DMF loaded did not show therapeutic effects on RAW264.7 or NIH-3T3 cells, which confirmed the therapeutic effect of DMF (Fig. 6 l, m). However, no obvious change in CD206 protein levels after DMF or NPs administration was found (Additional file 12: Fig. S12). Taken together, these results indicate that DTP@DMF NPs can inhibit the production of TGF-β by promoting the expression of Nrf2 and HO-1 in macrophages, thereby suppressing the fibroblast differentiation into myofibroblasts and collagen production.

### DTP@DMF NPs alleviate pulmonary fibrosis and M2 macrophage accumulation by activating Nrf2 signaling

**Fig. 7:**
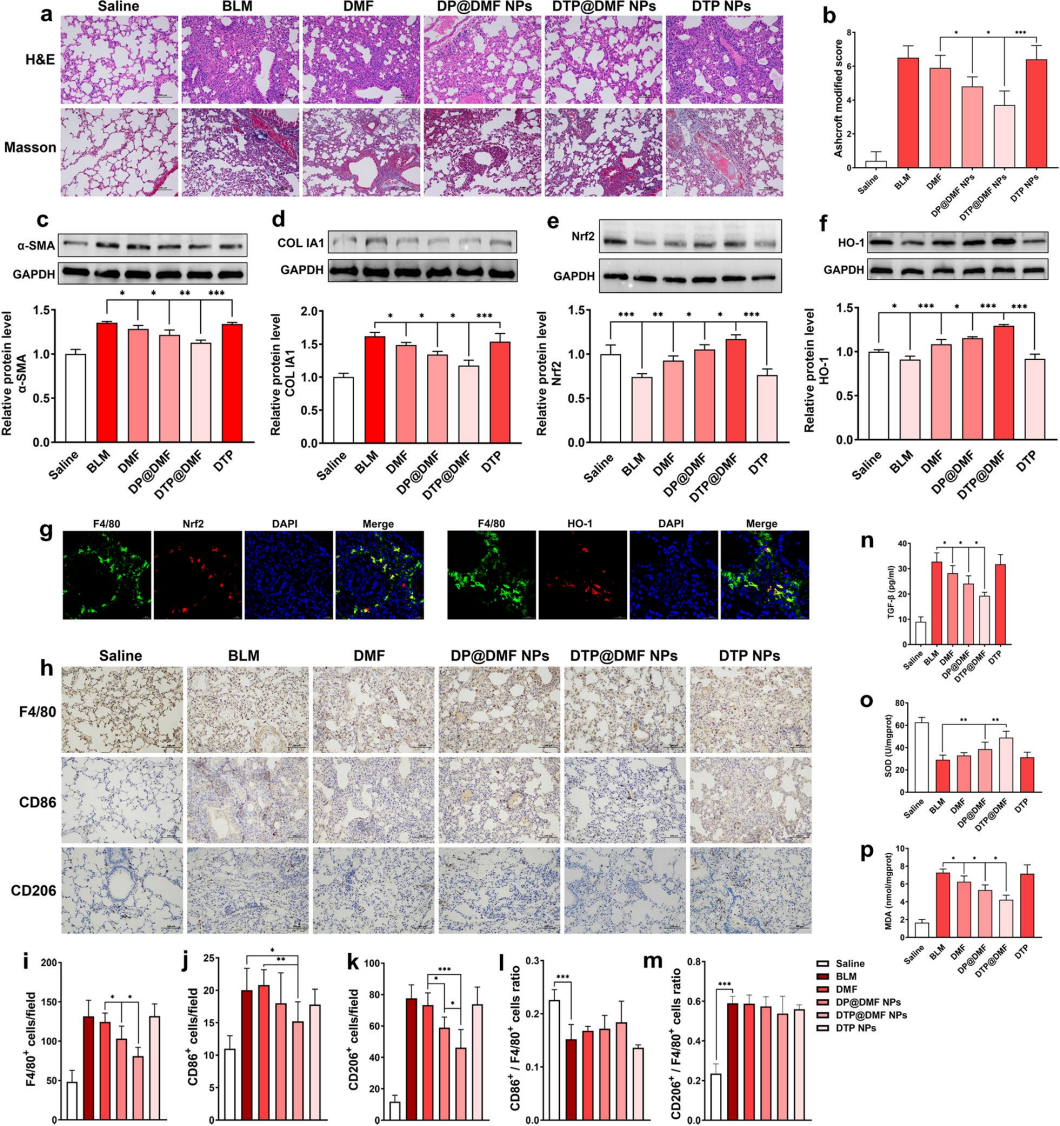
DTP@DMF NPs suppress fibrosis and macrophage accumulation in lung tissue via Nrf2 signaling. **a** Representative H&E and Masson’s trichrome staining and **b** the severity score of fibrosis after DMF, DP@DMF NPs and DTP@DMF NPs treatment (n = 5). **c** α-SMA and **d** collagen Ia1 protein levels in fibrotic tissue (n = 3). **e** Nrf2 and **f** HO-1 levels in fibrotic tissue (n = 3). **g** The Nrf2 (red) and HO-1 (red) expression in F4/80^+^ macrophages (green) detected by immunofluorescence analysis. **h** Immunohistochemistry of F4/80^+^, CD86^+^, and CD206^+^ macrophages in pulmonary tissues, and **i**–**m** quantification of total macrophages and M1 and M2 phenotypes (n = 5). **n** TGF-β levels in BALF, **o** SOD and **p** MDA levels in tissue (n = 5). Statistical analyses were performed via one-way ANOVA with S–N-K post hoc analysis. * *P* < 0.05, ** *P* < 0.01, *** *P* < 0.001

We compared the therapeutic effect of DTP@DMF NPs with other NPs on pulmonary fibrosis in vivo. Moreover, the mechanism of DTP@DMF NPs attenuate fibrosis was explored. As shown in Fig. 7a, b, the H&E staining and Masson’s trichrome staining indicated that the direct administration of DMF had a limited therapeutic effect on pulmonary fibrosis, while the intratracheal instillation of DMF-encapsulated DP@DMF NPs significantly improved the therapeutic effect on pulmonary fibrosis. Compared with DP@DMF NPs, DTP@DMF NPs further improved the therapeutic effect of pulmonary fibrosis, and pulmonary fibrosis was significantly reduced, as shown by H&E staining and Masson’s trichrome staining. Western blot detection of α-SMA and collagen Ia1 also revealed that DTP@DMF NPs could inhibit myofibroblast transformation and attenuate collagen Ia1 production more potently than DMF and DP@DMF NPs (Fig. 7c, d). Besides, the DTP@DMF NPs decreased the IL-4 and IL-13 concentrations in BALF (Additional file 13: Fig. S13), as well as reversing the body weight of mice with pulmonary fibrosis (Additional file 14: Fig. S14).

Furthermore, we examined the effects of various NPs on Nrf2 and HO-1 levels. The results showed that the levels of Nrf2 and HO-1 in fibrotic lung tissues were significantly decreased, while the upregulation of Nrf2 and HO-1 by DTP@DMF NPs was the most obvious (Fig. 7e, f). We confirmed by immunofluorescence that Nrf2 and HO-1 were highly expressed in macrophages in lung tissue (Fig. 7g). Therefore, we next compared the effect of different liposomes on activity of macrophages. Administration of DMF slightly reduced the total numbers of F4/80^+^ cells and CD206^+^ macrophages, while DP@DMF NPs more significantly reduced the total numbers of F4/80^+^ cells and CD206^+^ macrophages. Compared with DP@DMF NPs, DTP@DMF NPs had a more pronounced effect on activity of macrophages. However, no change in the CD206^+^ macrophage or CD86^+^ macrophage ratio was found (Fig. 7h–m). Since macrophages are one of the main cell types that generate TGF-β and ROS, we next analyzed the differences in the effects of various treatments on levels of TGF-β and ROS related products. The detection of TGF-β levels in BALF showed that DTP@DMF NPs had the most significant effect on reducing the level of TGF-β compared to direct administration of DMF and DP@DMF NPs (Fig. 7n). The detection of the oxidative stress related products SOD and MDA in lung tissue also revealed that the DTP@DMF NPs could more reduce the oxidative stress response (Fig. 7o, p).

In addition, we compared the therapeutic effect on fibrosis between inhalation administration of DTP@DMF NPs and intravenous DMF administration. Mice with pulmonary fibrosis were received DTP@DMF inhalation (DMF: 5 mg/kg) and DMF intravenous administration (DMF: 50 mg/kg). As shown in Additional file 15: Fig. S15, although the dose of DMF through intravenous injection is 10 times that of inhalation, we found that the inhalation administration of DMF-encapsuled liposomes exerted more powerful suppressive effect on pulmonary fibrosis. Besides, inhalation therapy promoted the Nrf2 and HO-1 expression than intravenous administration of DMF (Additional file 16: Fig. S16). The above results show that inhaled liposomes have advantages over traditional administration of DMF. Moreover, the direct intratracheal administration of DMF has a limited effect in the treatment of pulmonary fibrosis, while the ROS-responsive liposome DTP@DMF NPs inhalation can attenuate the accumulation of macrophages at the site of fibrosis and inhibit the generation of TGF-β and ROS by upregulating the Nrf2 signaling, thereby alleviating pulmonary fibrosis.

### In vivo biodistribution and biosafety of DTP@DMF NPs

**Fig. 8:**
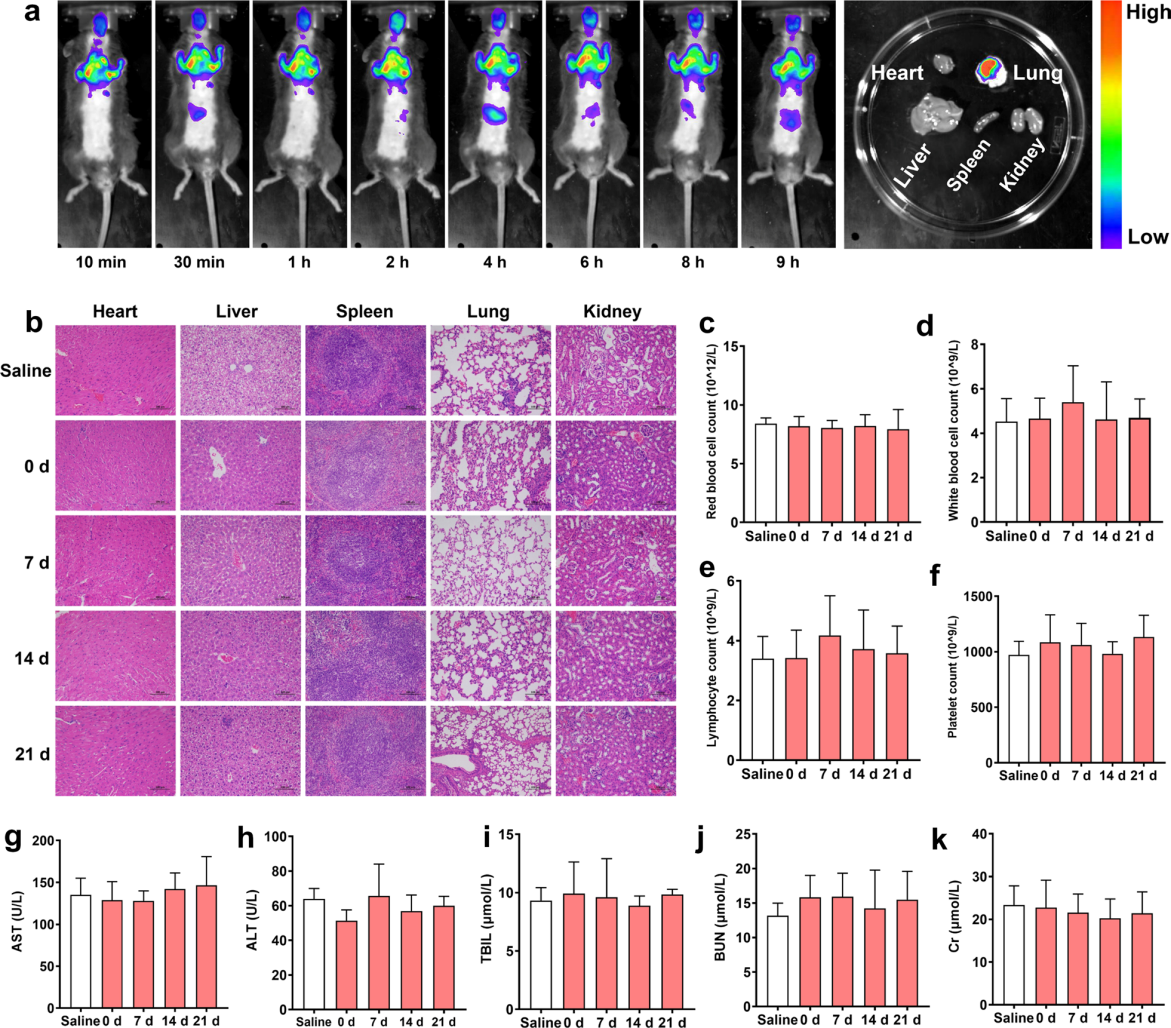
In vivo biodistribution and biosafety of DTP@DMF NPs. **a** The fluorescence images of mice at different timepoints after DiR-labeled DTP@DMF NPs intratracheal instillation and the fluorescence intensity of major organs after 9 h. **b** The H&E staining of major organs of mice after DTP@DMF NPs interference. **c**–**k** The blood test of mice receiving DTP@DMF NPs. Statistical analyses were performed via one-way ANOVA with S–N-K post hoc analysis (n = 5). * *P* < 0.05, ** *P* < 0.01, *** *P* < 0.001

To clarify the biodistribution of DTP@DMF NPs in mice, DiR-loaded NPs were prepared and administered intratracheally to mice with pulmonary fibrosis. Then, the mice were taken in vivo fluorescence imaging at 10 min, 30 min, 1 h, 2 h, 4 h, 6 h, 8 h, and 9 h after administration. As demonstrated in Fig. 8a, fluorescent NPs accumulated in the lung for a long time, and the fluorescence signal only slightly weakened with the prolongation of time. After 9 h, we sacrificed the mice and removed the main organs of the mice for fluorescence signal collection in vitro. Consistent with the results of in vivo fluorescence signal analysis, in vitro fluorescence images showed that the liposomes were mainly concentrated in the lungs after intratracheal instillation and were minimally distributed in other organs, such as the liver and kidney. Furthermore, the potential toxicity of NPs was evaluated to validate the biocompatibility and biosafety of DTP@DMF NPs in C57BL/6 mice. There were no histopathological lesions found in H&E staining of major organs in mice after 0, 7, 14, and 21 days (Fig. 8b). Furthermore, the results of routine blood test and biochemical factors of the liver and kidneys demonstrated the good biosafety of DTP@DMF NPs, indicating that the NPs have the potential clinical application prospect. (Fig. 8c–k).

## Conclusions

In this study, we synthesized ROS-responsive liposome-DTP@DMF NPs. DTP@DMF NPs have an ideal ability to release the drug in a ROS-enriched microenvironment. The progression of pulmonary fibrosis is closely related to the macrophage aggregation and M2-type polarization of macrophages, as well as the induction of TGF-β and ROS. DTP@DMF NPs can reduce macrophage accumulation and suppress the production of TGF-β and ROS to reduce fibroblast-to-myofibroblast transition and ECM deposition, thereby attenuating the progression of pulmonary fibrosis. These effects are accomplished by upregulating Nrf2 and HO-1. Moreover, DTP@DMF NPs can accumulate in the lung tissue for a long time, minimizing the exposure of the drug in the whole body, and have a very good biocompatibility. Our study proposed that ROS-responsive liposome is an ideal delivery system for inhaled drugs and have a better therapeutic effect than direct drug inhalation on pulmonary fibrosis treatment. Our study also revealed that DMF is effective for treating pulmonary fibrosis. Since DMF has been approved by the FDA for clinical application, the DTP@DMF NPs has a good translational potential for the treatment of pulmonary fibrosis in the future.

## Supplementary Information

**Additional file 1: Fig. S1.** The appearance change of liposome solutions within 7 days. From left to right: DP@DMF NPs, DTP@DMF NPs, DTP NPs.

**Additional file 2: Fig. S2.** The UV–Vis-NIR absorption spectra of DMF, DP@DMF NPs, DTP@DMF NPs, and DTP NPs.

**Additional file 3: Fig. S3.** The UV–Vis-NIR absorption spectra of DMF at different concentrations.

**Additional file 4: Fig. S4.** The TEM images of DTP@DMF NPs after incubating with H_2_O_2_ (100 μM) for 9 h. scale bar: 500 nm.

**Additional file 5: Fig. S5.** The DMF release profile of DTP@DMF NPs in the presence and absence of H_2_O_2_.

**Additional file 6: Fig. S6.** The body weight change of mice during fibrosis development (n = 5).

**Additional file 7: Fig. S7.** The IL-4 and IL-13 levels in BALF of mice after DTP@DMF NPs treatment with various concentrations (n = 5). * *P* < 0.05, ** *P* < 0.01.

**Additional file 8: Fig. S8.** The body weight change of mice after DTP@DMF NPs treatment with various concentrations (n = 5).

**Additional file 9: Fig. S9.** IL-4 and IL-13 levels in BALF after DTP@DMF NPs treatment at different phases of fibrosis. * *P* < 0.05, ** *P* < 0.01.

**Additional file 10: Fig. S10.** The cellular endocytosis efficacy of DP@DMF NPs and DTP@DMF NPs. Flow analysis of cellular endocytosis of DiI-labeled NPs in RAW264.7 cells at 0, 2 and 4 h. The V1R represented the percentage of RAW264.7 cells which engulfed DiI-labeled liposomes.

**Additional file 11: Fig. S11.** SOD and MDA levels in RAW264.7 cells after interference of DMF and NPs (n = 5). * *P* < 0.05, ** *P* < 0.01, *** *P* < 0.001.

**Additional file 12: Fig. S12.** The CD206 protein expression of RAW264.7 cells receiving IL-4 interference and DTP@DMF NPs treatment (n = 3).

**Additional file 13: Fig. S13.** The IL-4 and IL-13 levels in BALF after DMF and NPs treatment (n = 5). * *P* < 0.05.

**Additional file 14: Fig. S14.** The body weight change of mice receiving the DMF and NPs treatment (n = 5).

**Additional file 15: Fig. S15.** The therapeutic effects of DTP@DMF NPs inhalation and DMF intravenous administration on pulmonary fibrosis. (a) H&E and Masson’s trichrome staining and (b) the modified Ashcroft score of pulmonary fibrosis (n = 5). (c) α-SMA and (d) collagen Ia1 protein levels in fibrotic tissue (n = 3). * *P* < 0.05, ** *P* < 0.01.

**Additional file 16: Fig. S16.** The effects of DTP@DMF NPs inhalation and DMF intravenous administration on Nrf2 signaling in lung tissue. (a) The Nrf2 and (b) HO-1 expression in lung tissue (n = 3). * *P* < 0.05, ** *P* < 0.01, *** *P* < 0.001.

## Data Availability

The datasets used and/or analysed during the current study are available from the corresponding author on reasonable request.

## Abbreviations

IPF: Idiopathic pulmonary fibrosis
TGF-β: Transforming growth factor-β
ROS: Reactive oxygen species
Nrf2: Nuclear factor erythroid 2 related factor 2
DMF: Dimethyl fumarate
HO-1: Heme oxygenase-1
NPs: Nanoparticles
ECM: Excessive extracellular matrix
IL: Interleukin
PDGF: Platelet derived growth factor
Keap1: Kelch-like ECH-associated protein 1
ARE: Antioxidant response elements
FDA: Food and Drug Administration
TK: Thioketal
PFOB: Perfluorooctyl bromide
BLM: Bleomycin
TEM: Transmission electron microscopy
NMR: Nuclear magnetic resonance
EE: Encapsulation efficiency
DLC: Drug loading capacity
SOD: Superoxide dismutase
MDA: Malondialdehyde
UV–Vis-NIR: Ultraviolet–visible-near infrared
H&E: Hematoxylin and eosin
Fig.: Figure
BALF: Bronchoalveolar lavage fluid
SMA: Smooth muscle actin
