# Supplementary figures and images for: ROS-responsive liposomes as an inhaled drug delivery nanoplatform for idiopathic pulmonary fibrosis treatment via Nrf2 signaling
Source: J Nanobiotechnology. 2022 May 6;20:213. doi: 10.1186/s12951-022-01435-4 (PMC9074278; doi:10.1186/s12951-022-01435-4)

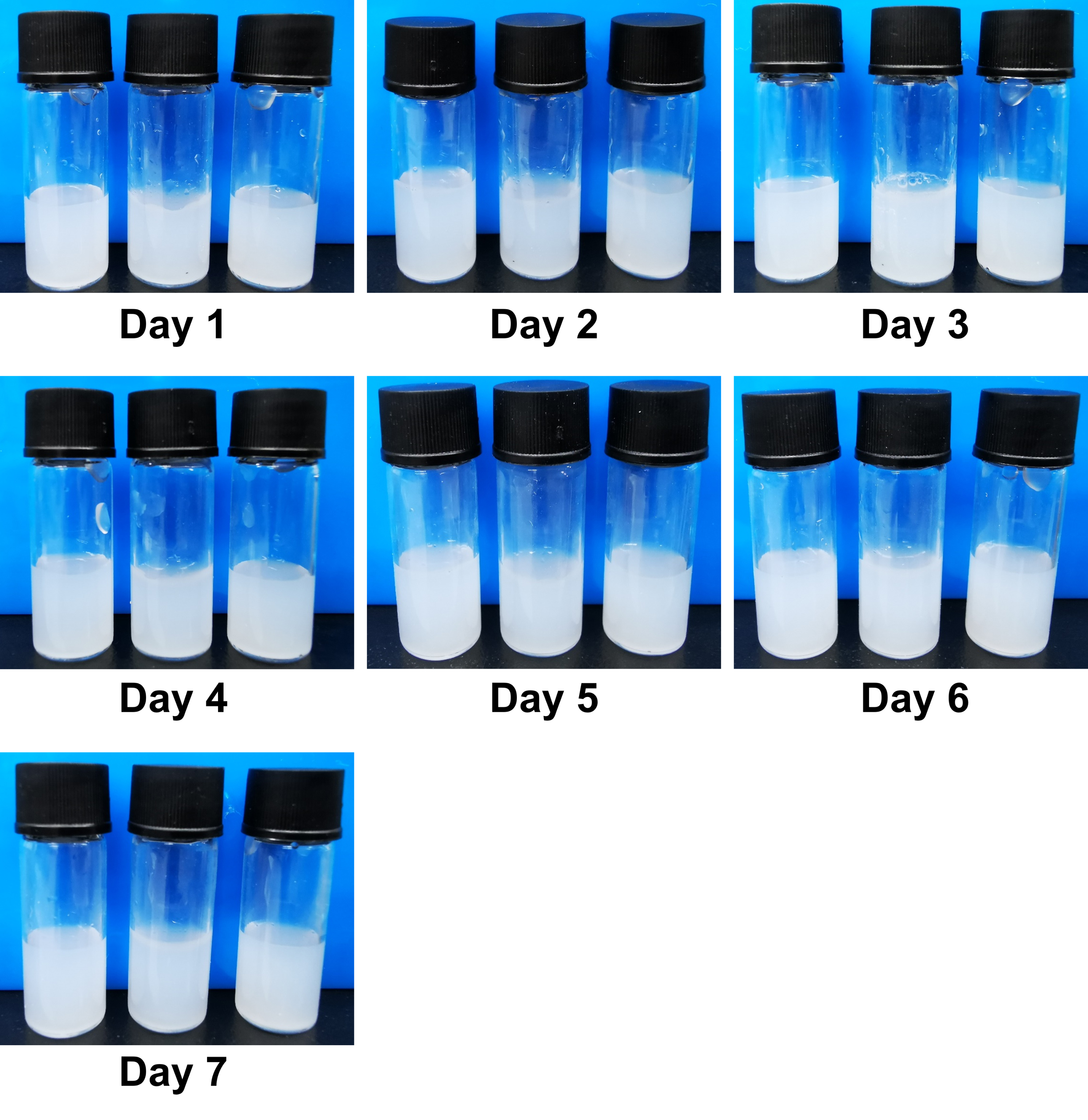

Supplement: Supplementary file 1 — Additional file 1: Fig. S1. The appearance change of liposome solutions within 7 days. From left to right: DP@DMF NPs, DTP@DMF NPs, DTP NPs. [file 12951_2022_1435_MOESM1_ESM.jpg]

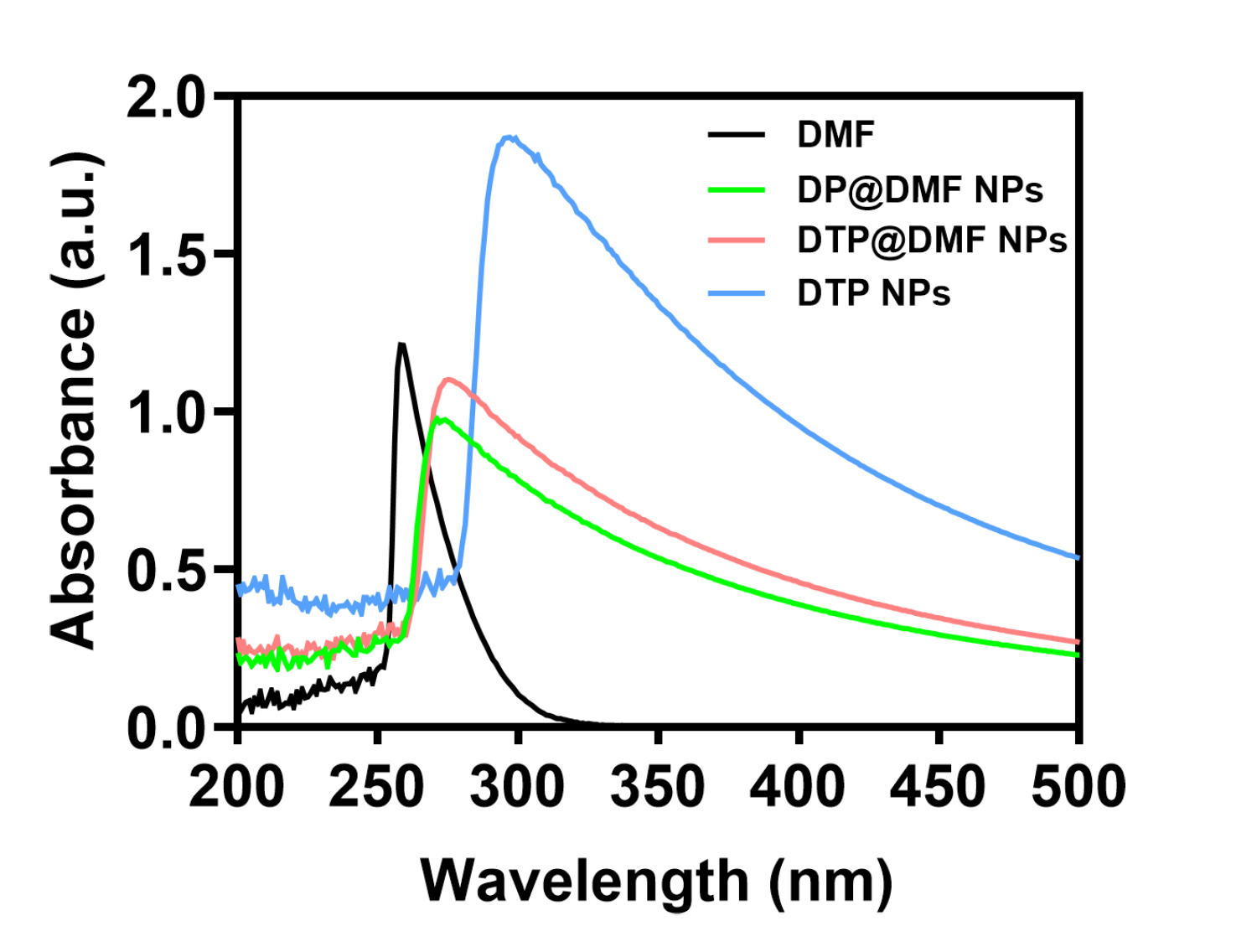

Supplement: Supplementary file 2 — Additional file 2: Fig. S2. The UV–Vis-NIR absorption spectra of DMF, DP@DMF NPs, DTP@DMF NPs, and DTP NPs. [file 12951_2022_1435_MOESM2_ESM.jpg]

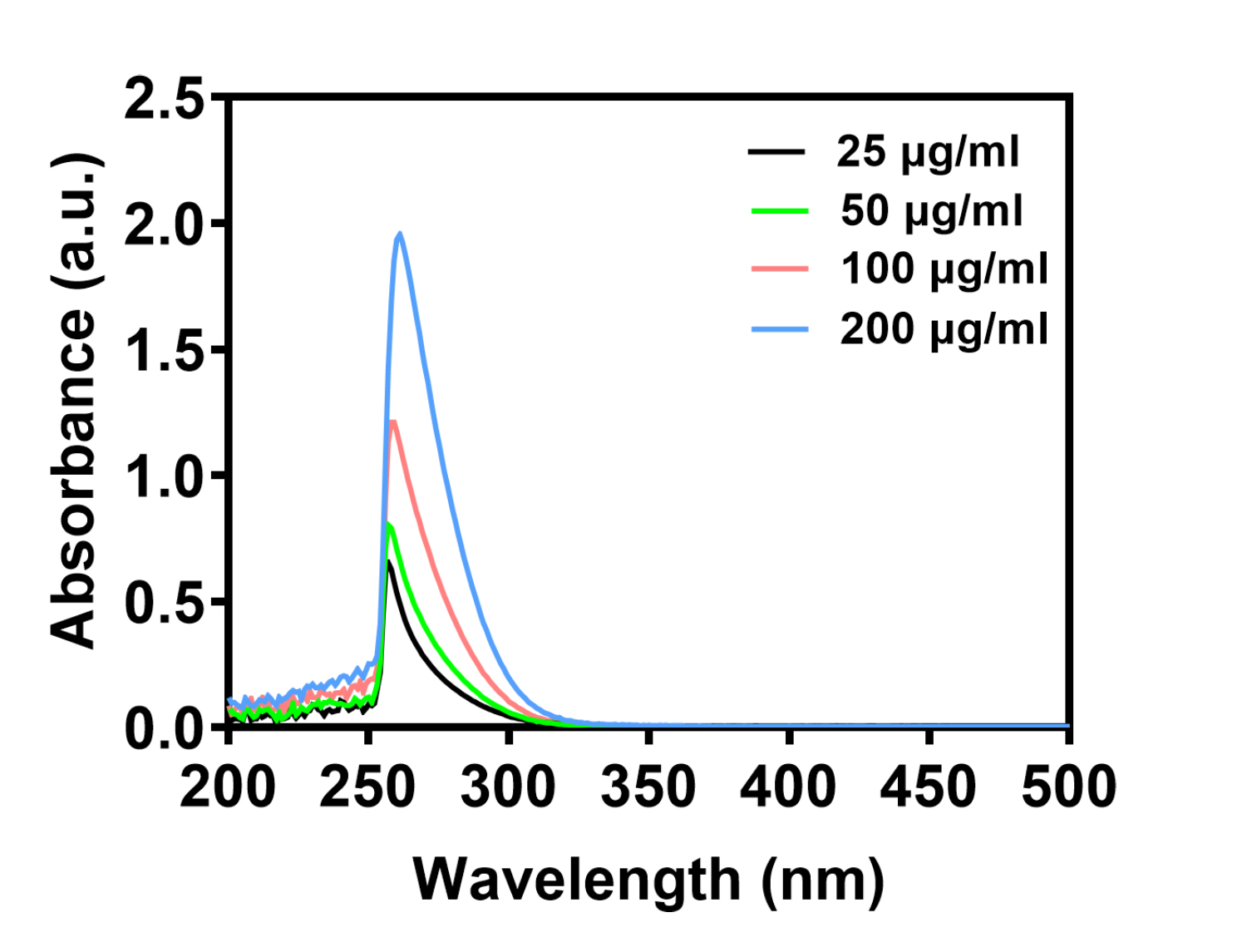

Supplement: Supplementary file 3 — Additional file 3: Fig. S3. The UV–Vis-NIR absorption spectra of DMF at different concentrations. [file 12951_2022_1435_MOESM3_ESM.jpg]

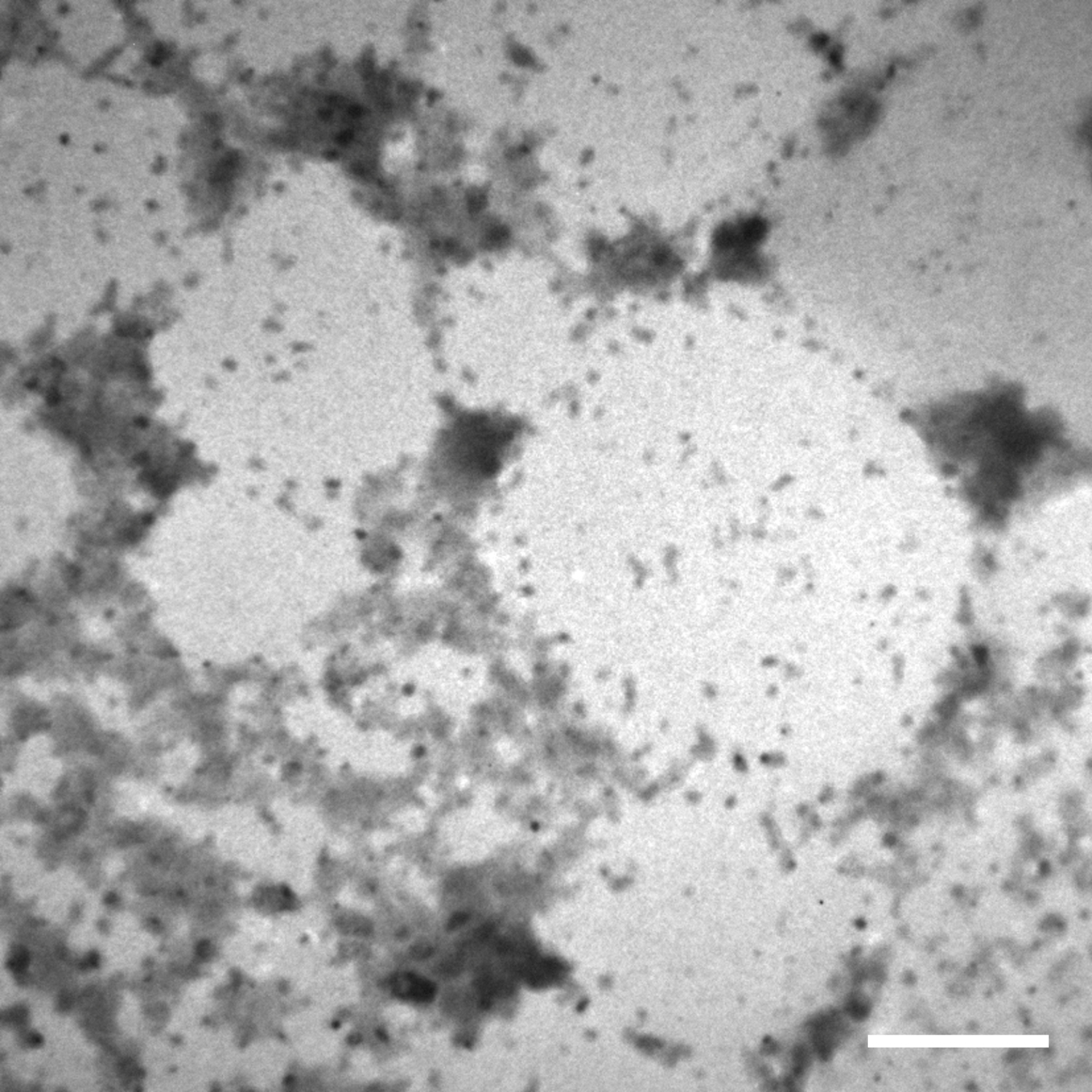

Supplement: Supplementary file 4 — Additional file 4: Fig. S4. The TEM images of DTP@DMF NPs after incubating with H2O2 (100 μM) for 9 h. scale bar: 500 nm. [file 12951_2022_1435_MOESM4_ESM.jpg]

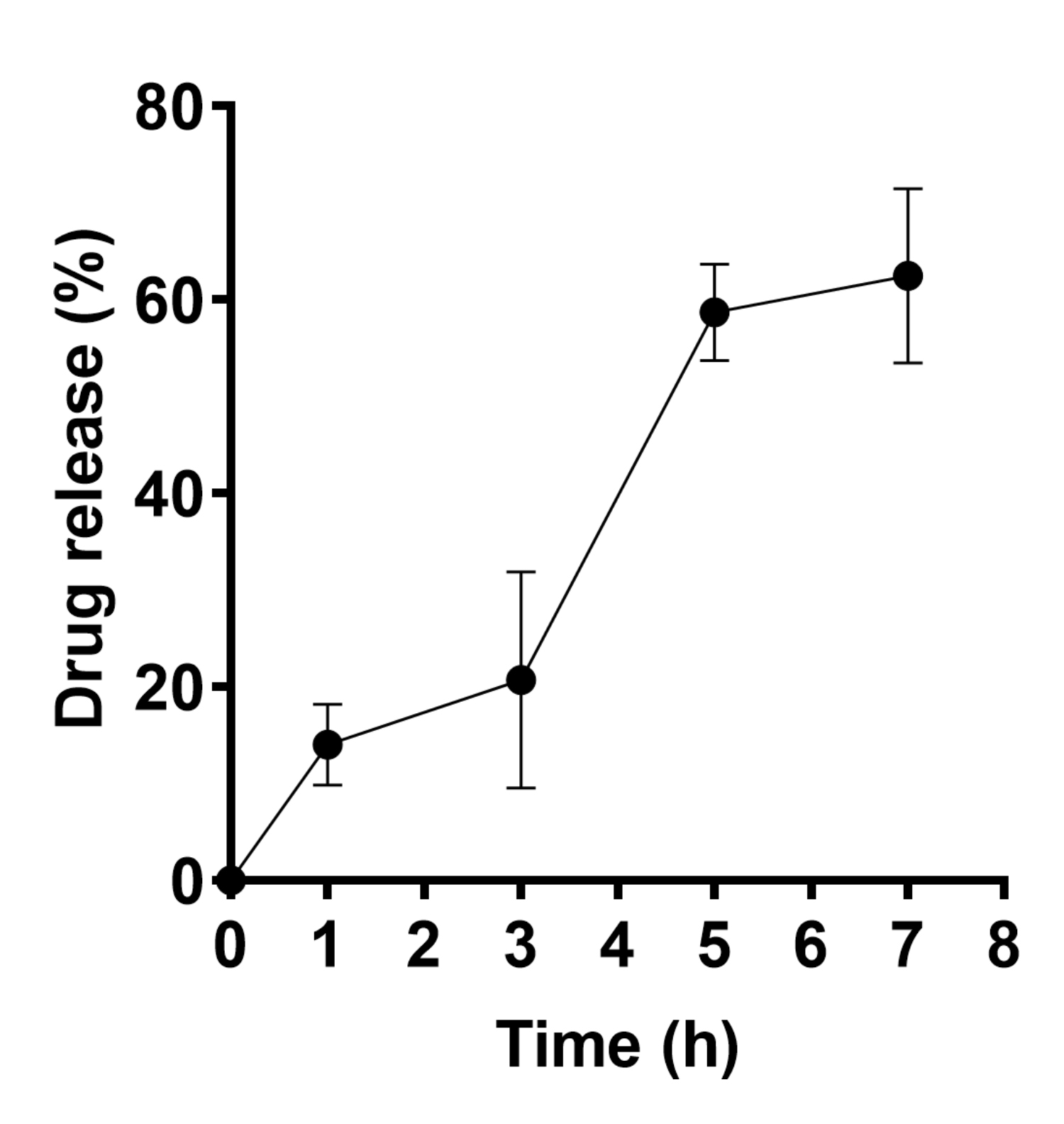

Supplement: Supplementary file 5 — Additional file 5: Fig. S5. The DMF release profile of DTP@DMF NPs in the presence and absence of H2O2. [file 12951_2022_1435_MOESM5_ESM.jpg]

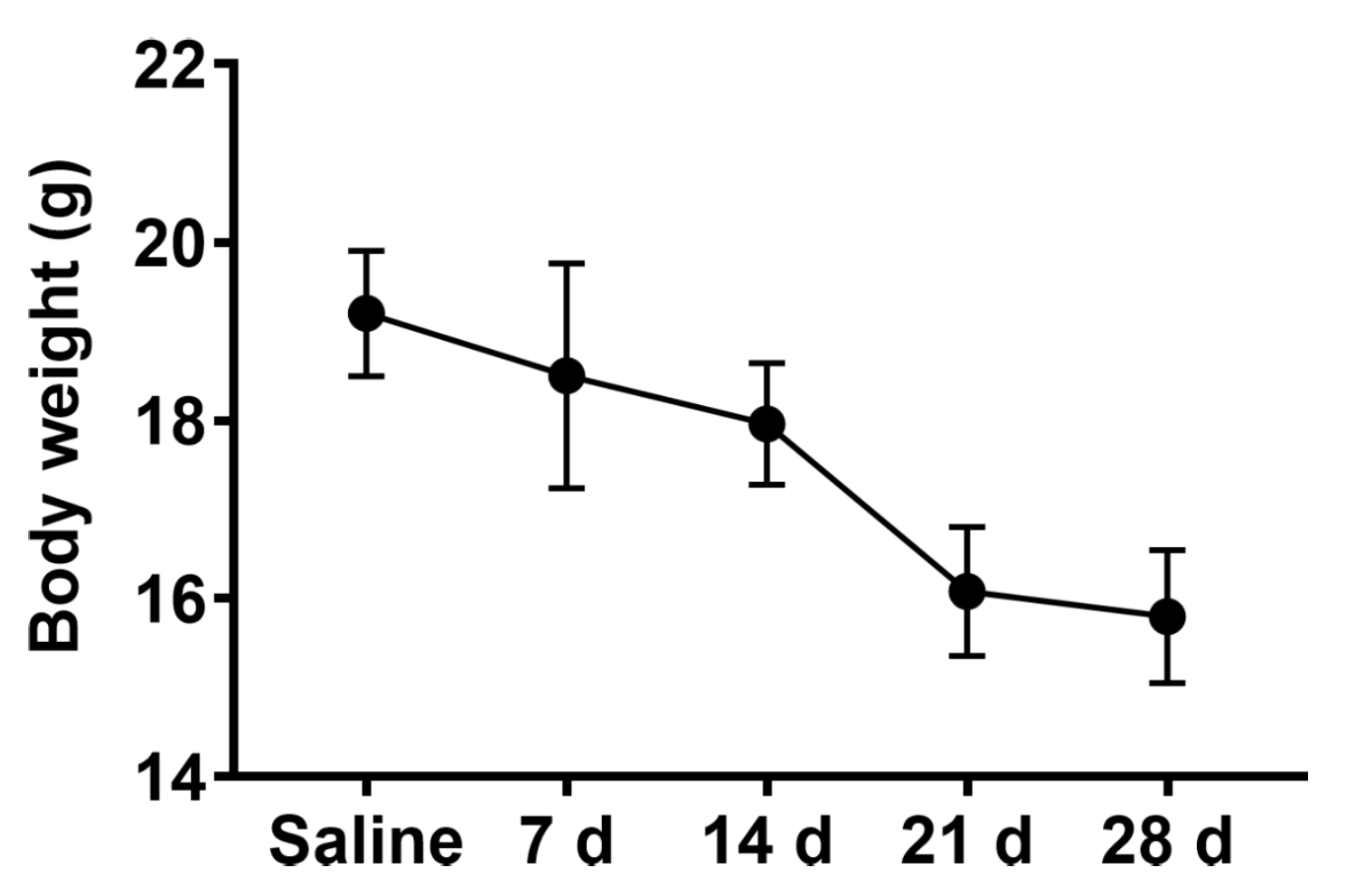

Supplement: Supplementary file 6 — Additional file 6: Fig. S6. The body weight change of mice during fibrosis development (n = 5). [file 12951_2022_1435_MOESM6_ESM.jpg]

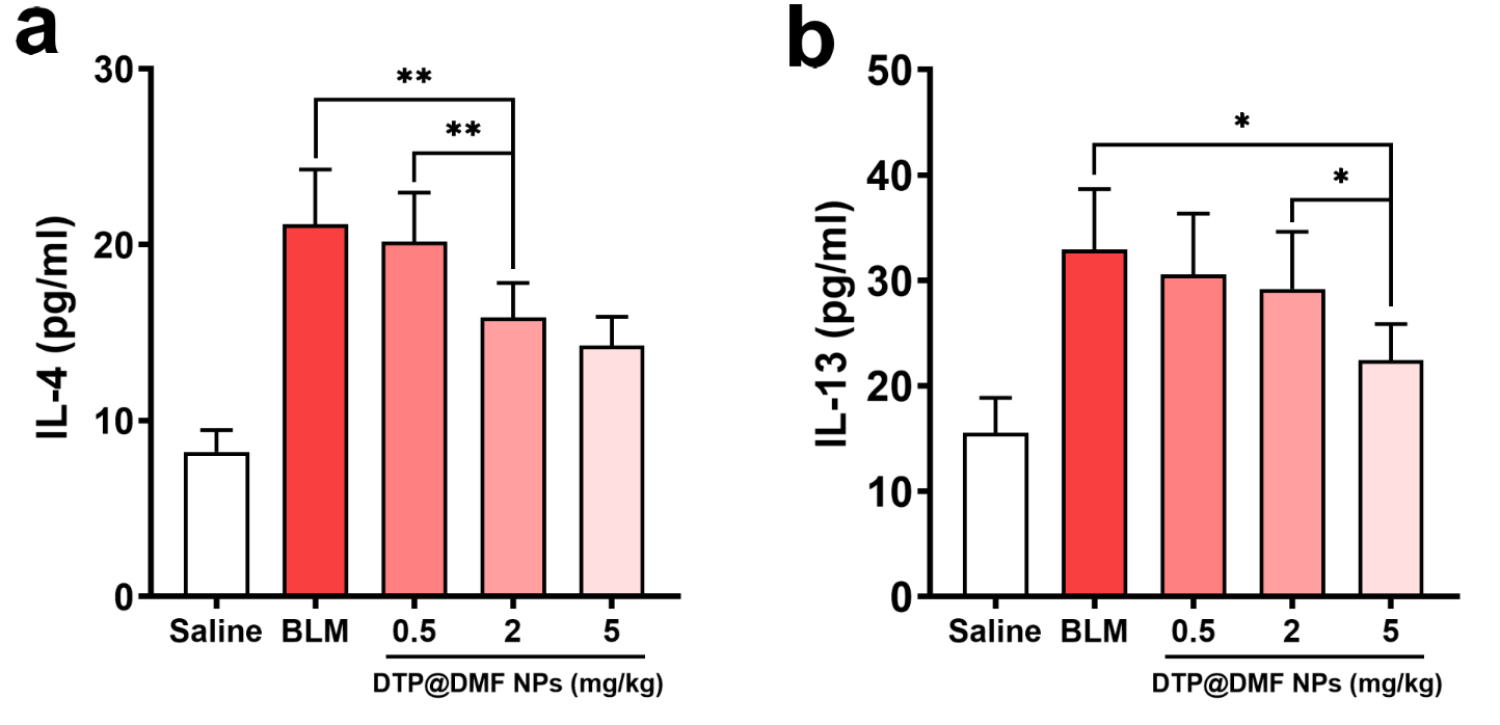

Supplement: Supplementary file 7 — Additional file 7: Fig. S7. The IL-4 and IL-13 levels in BALF of mice after DTP@DMF NPs treatment with various concentrations (n = 5). * P < 0.05, ** P < 0.01. [file 12951_2022_1435_MOESM7_ESM.jpg]

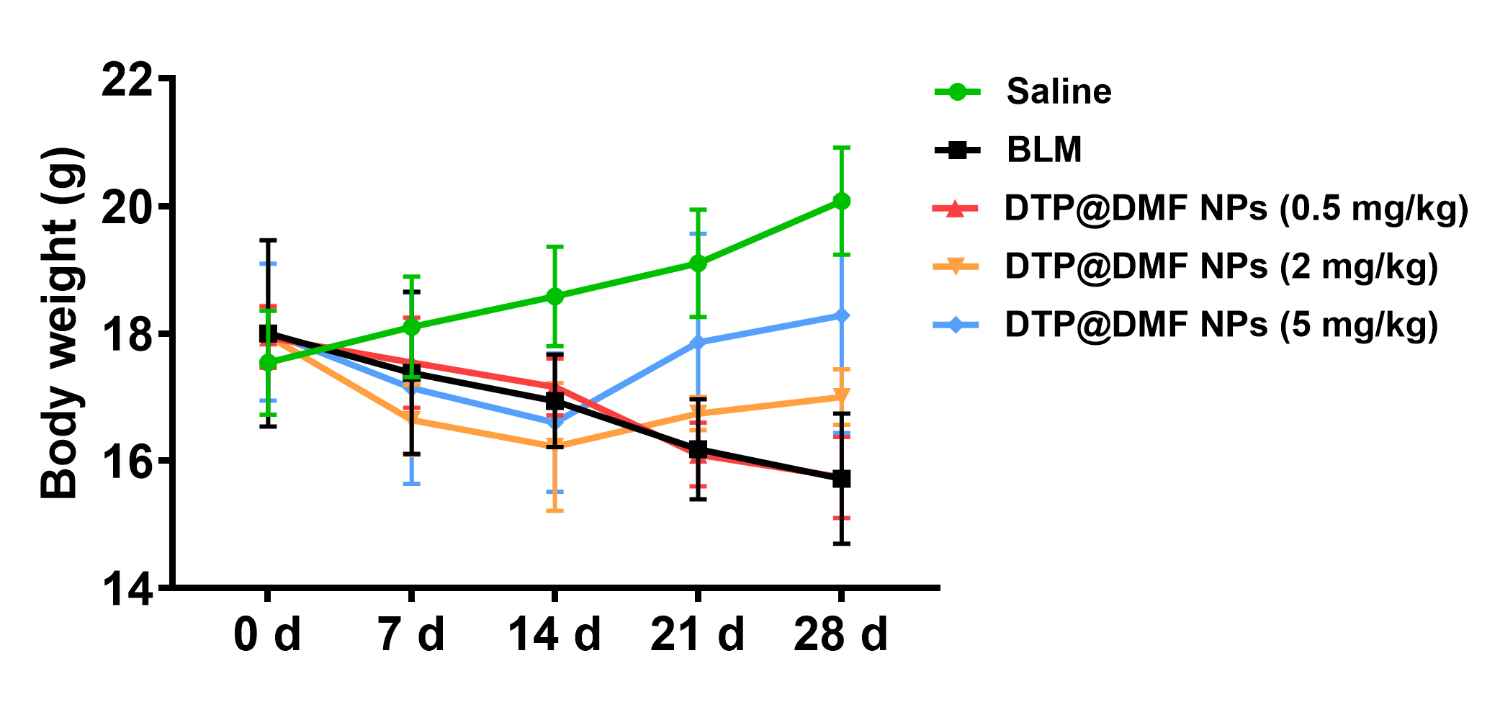

Supplement: Supplementary file 8 — Additional file 8: Fig. S8. The body weight change of mice after DTP@DMF NPs treatment with various concentrations (n = 5). [file 12951_2022_1435_MOESM8_ESM.jpg]

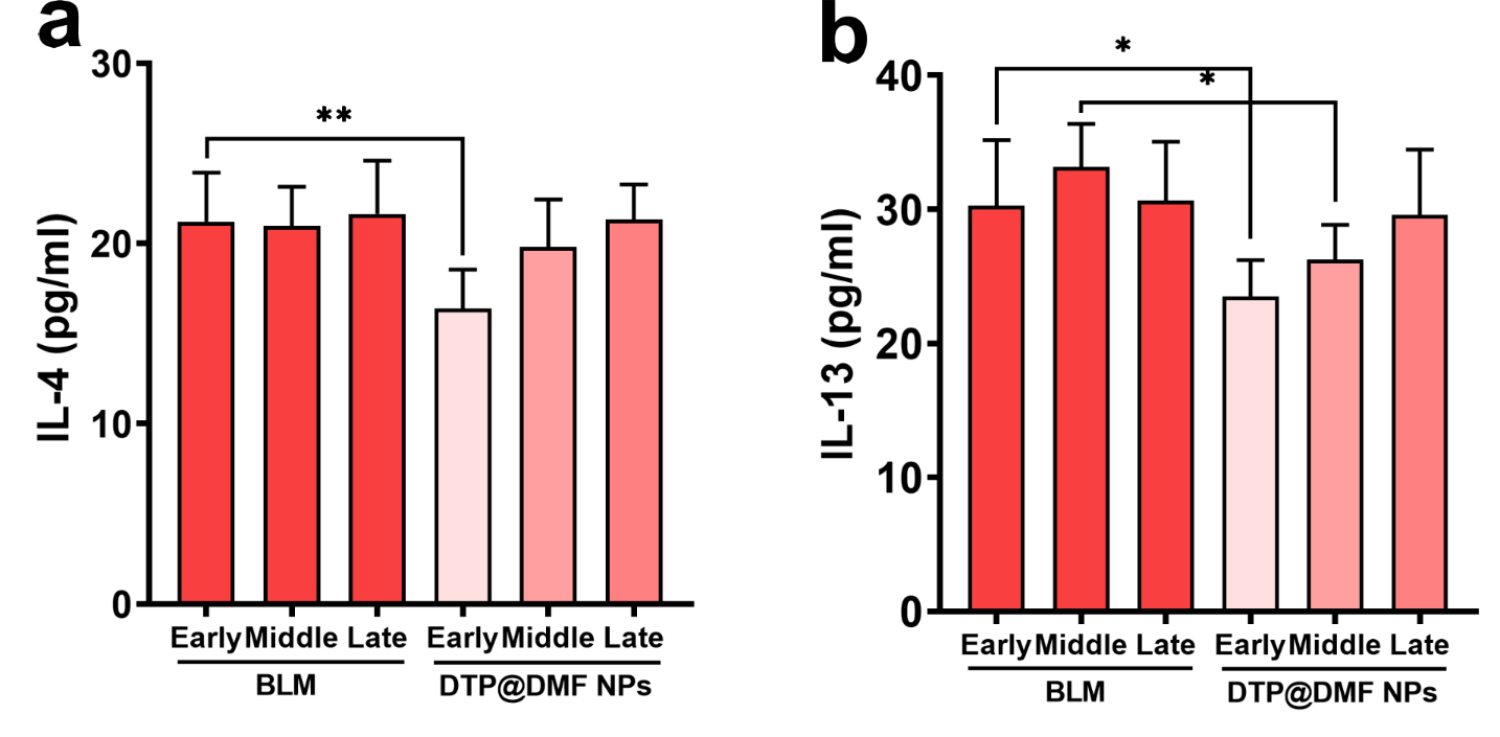

Supplement: Supplementary file 9 — Additional file 9: Fig. S9. IL-4 and IL-13 levels in BALF after DTP@DMF NPs treatment at different phases of fibrosis. * P < 0.05, ** P < 0.01. [file 12951_2022_1435_MOESM9_ESM.jpg]

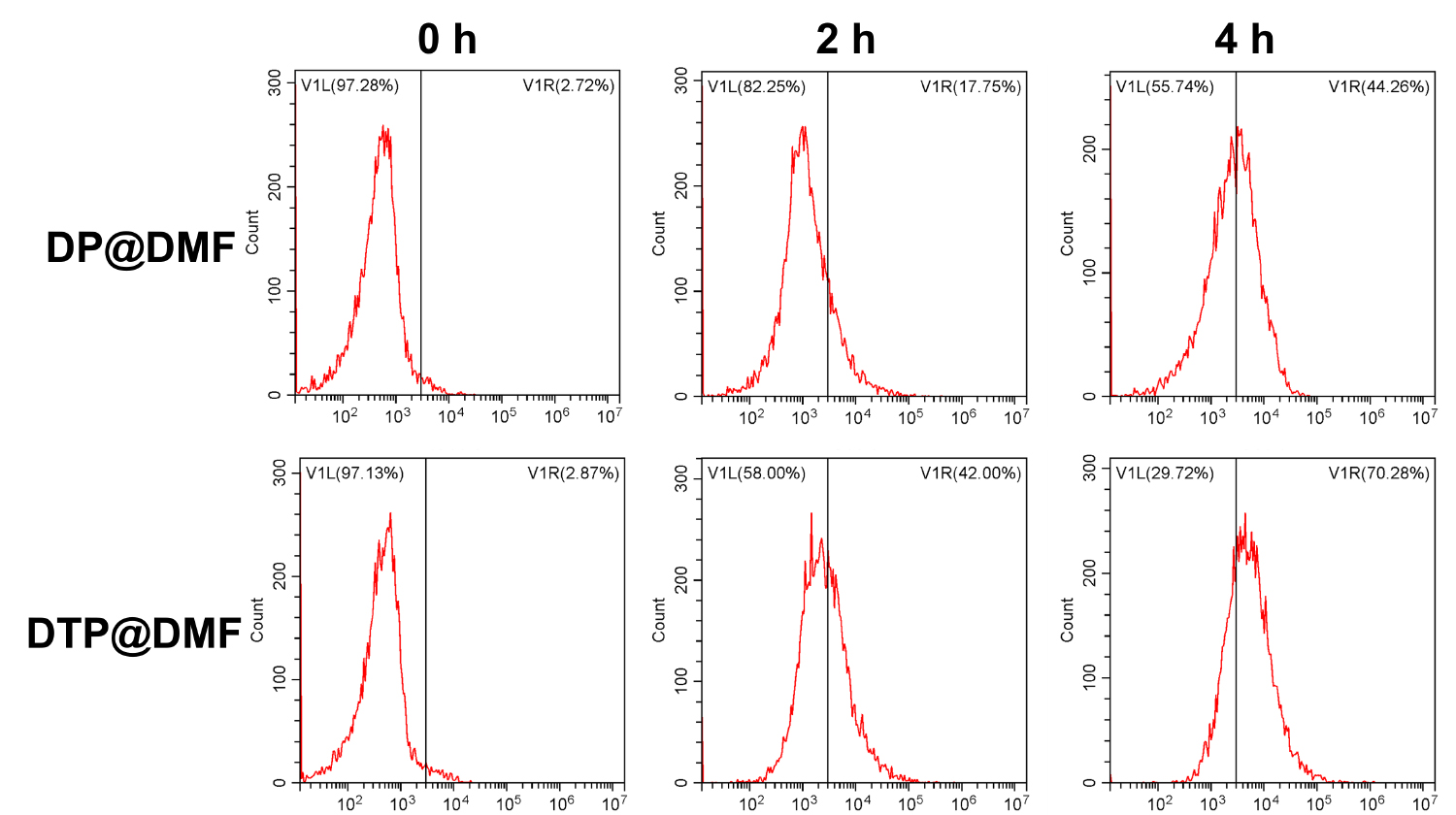

Supplement: Supplementary file 10 — Additional file 10: Fig. S10. The cellular endocytosis efficacy of DP@DMF NPs and DTP@DMF NPs. Flow analysis of cellular endocytosis of DiI-labeled NPs in RAW264.7 cells at 0, 2 and 4 h. The V1R represented the percentage of RAW264.7 cells which engulfed DiI-labeled liposomes. [file 12951_2022_1435_MOESM10_ESM.jpg]

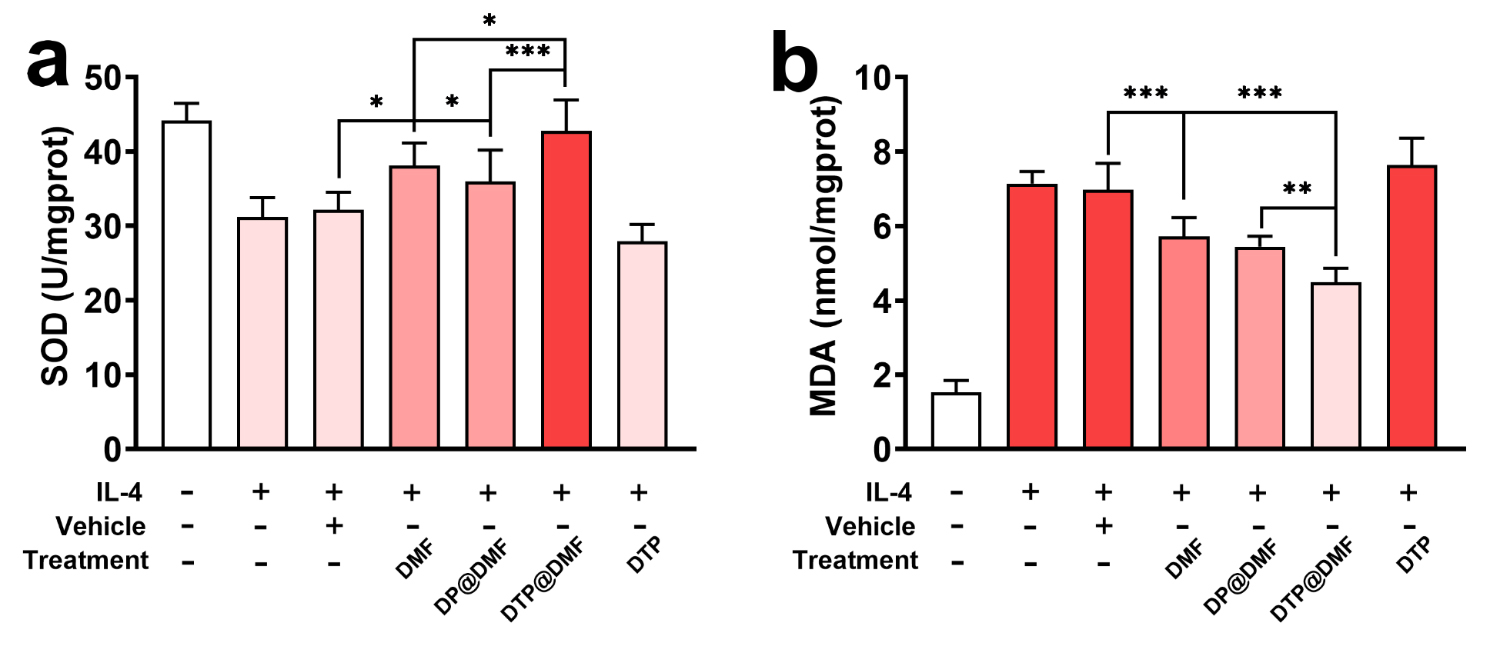

Supplement: Supplementary file 11 — Additional file 11: Fig. S11. SOD and MDA levels in RAW264.7 cells after interference of DMF and NPs (n = 5). * P < 0.05, ** P < 0.01, *** P < 0.001. [file 12951_2022_1435_MOESM11_ESM.jpg]

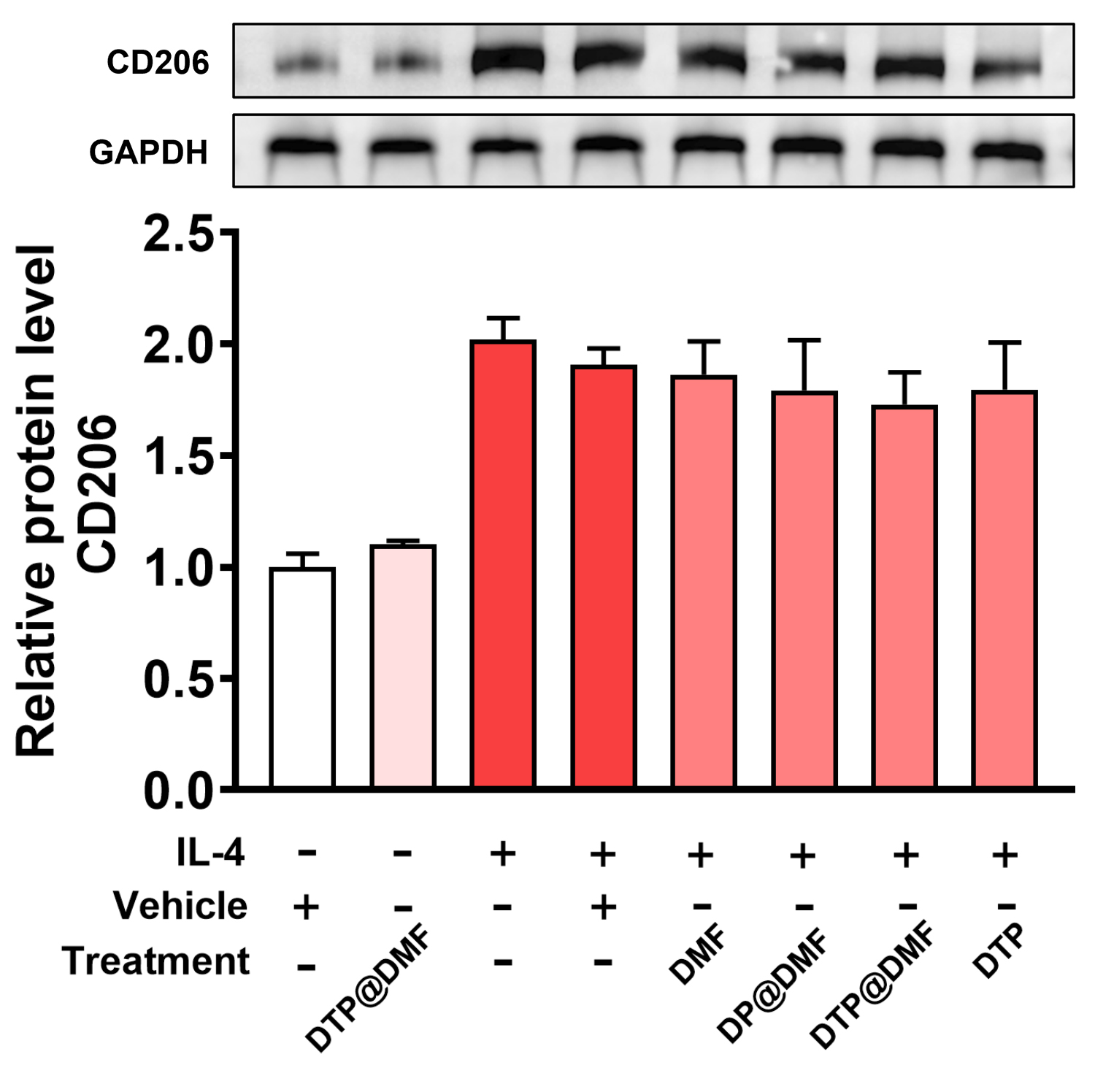

Supplement: Supplementary file 12 — Additional file 12: Fig. S12. The CD206 protein expression of RAW264.7 cells receiving IL-4 interference and DTP@DMF NPs treatment (n = 3). [file 12951_2022_1435_MOESM12_ESM.jpg]

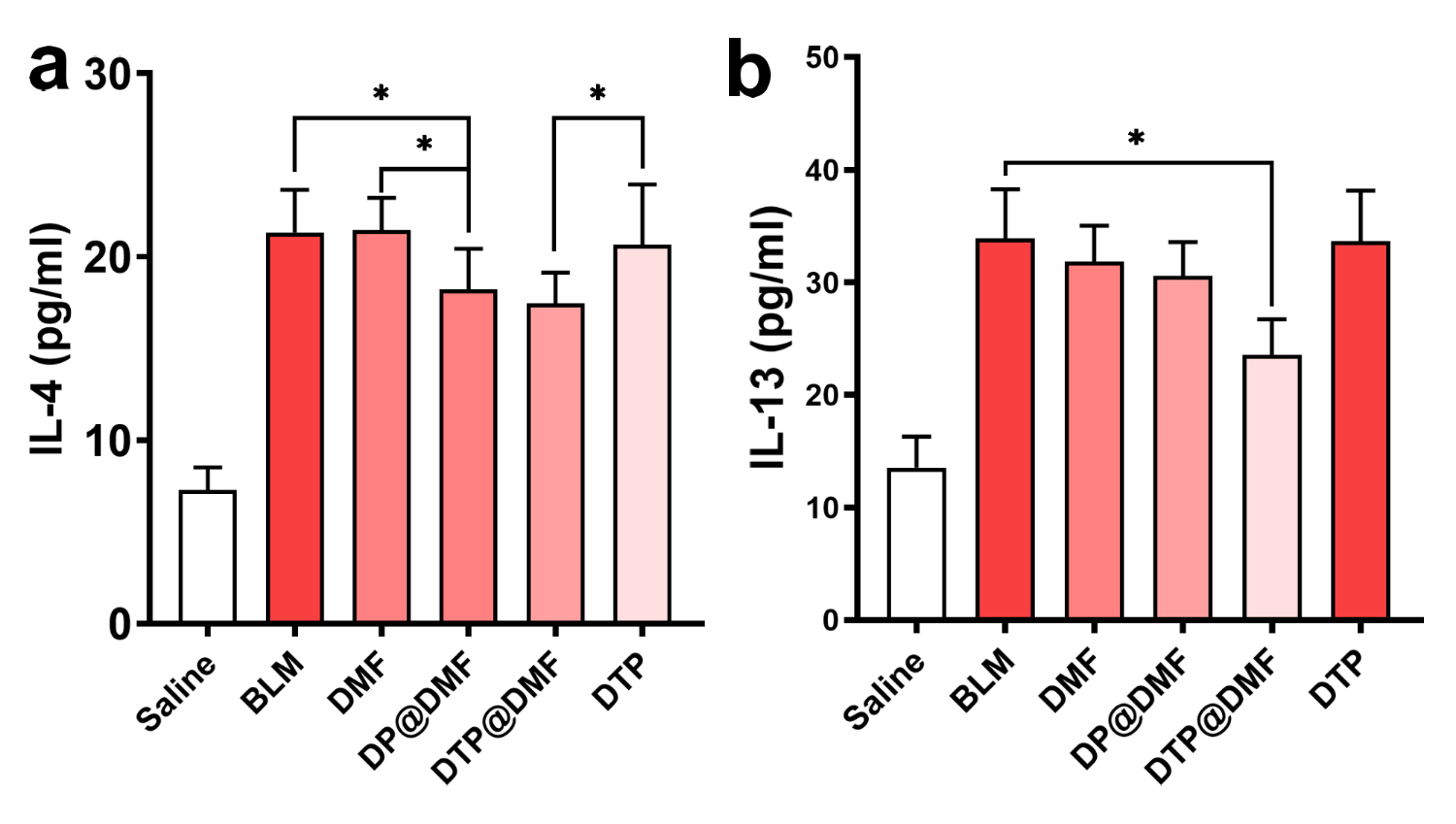

Supplement: Supplementary file 13 — Additional file 13: Fig. S13. The IL-4 and IL-13 levels in BALF after DMF and NPs treatment (n = 5). * P < 0.05. [file 12951_2022_1435_MOESM13_ESM.jpg]

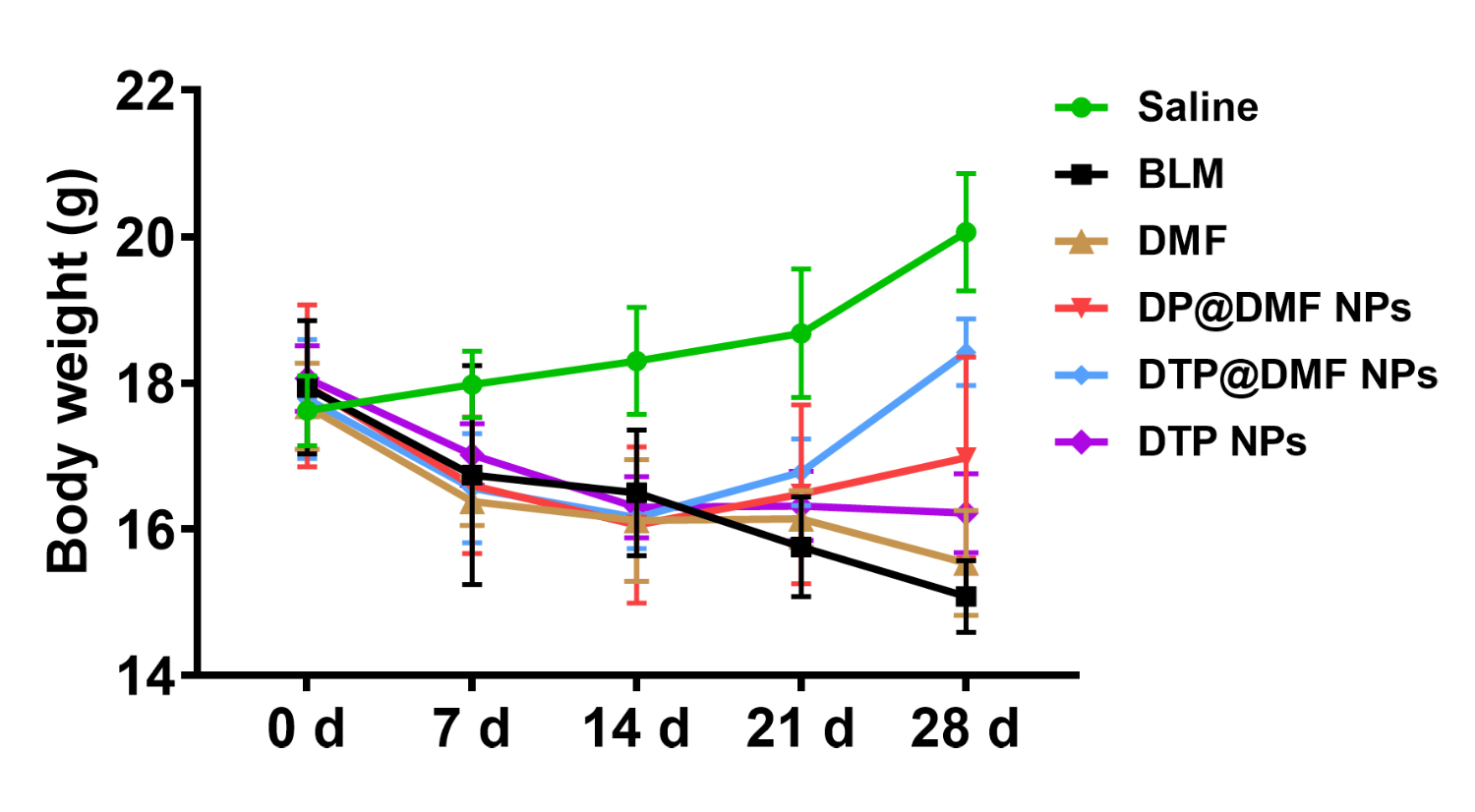

Supplement: Supplementary file 14 — Additional file 14: Fig. S14. The body weight change of mice receiving the DMF and NPs treatment (n = 5). [file 12951_2022_1435_MOESM14_ESM.jpg]

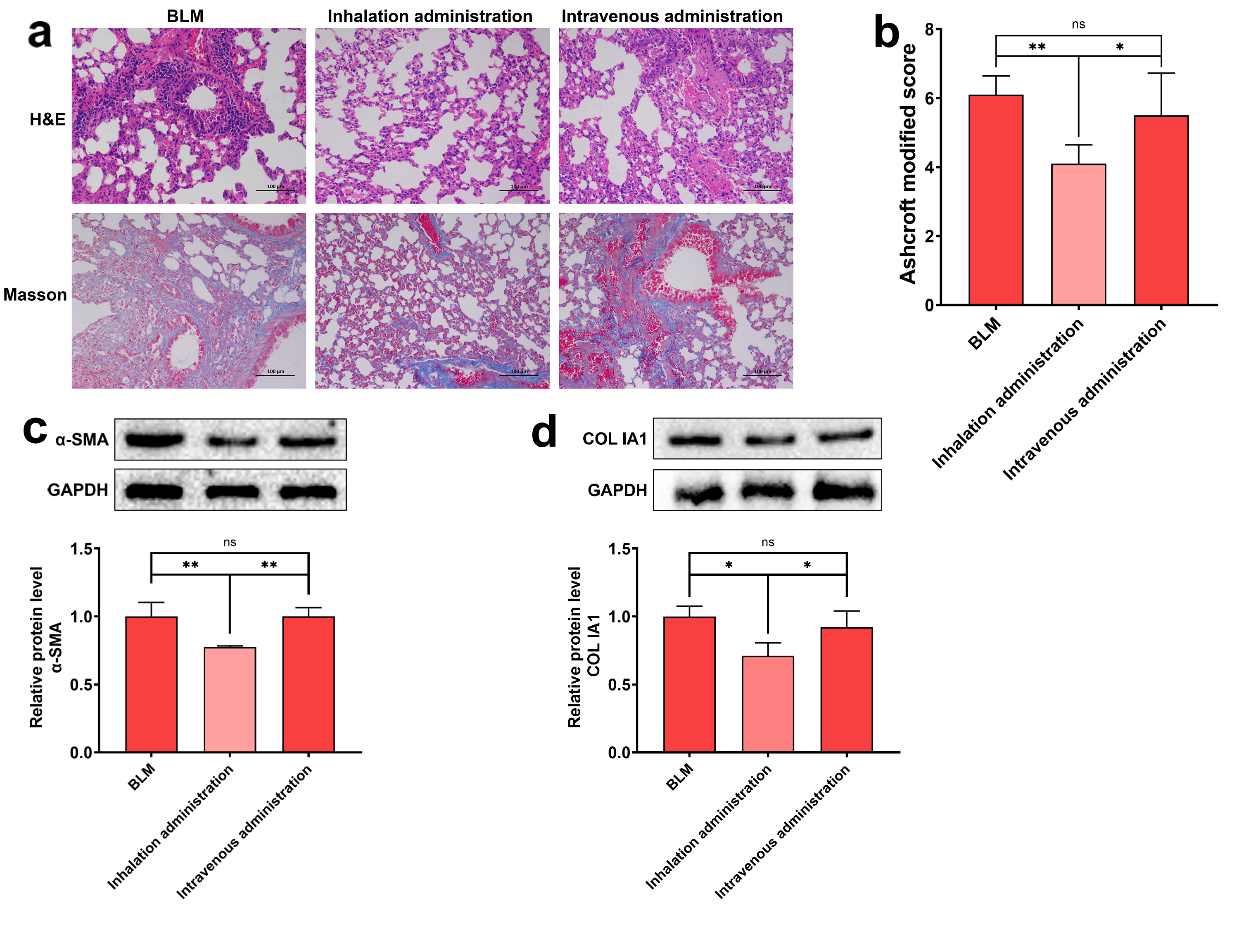

Supplement: Supplementary file 15 — Additional file 15: Fig. S15. The therapeutic effects of DTP@DMF NPs inhalation and DMF intravenous administration on pulmonary fibrosis. (a) H&E and Masson’s trichrome staining and (b) the modified Ashcroft score of pulmonary fibrosis (n = 5). (c) α-SMA and (d) collagen Ia1 protein levels in fibrotic tissue (n = 3). * P < 0.05, ** P < 0.01. [file 12951_2022_1435_MOESM15_ESM.jpg]

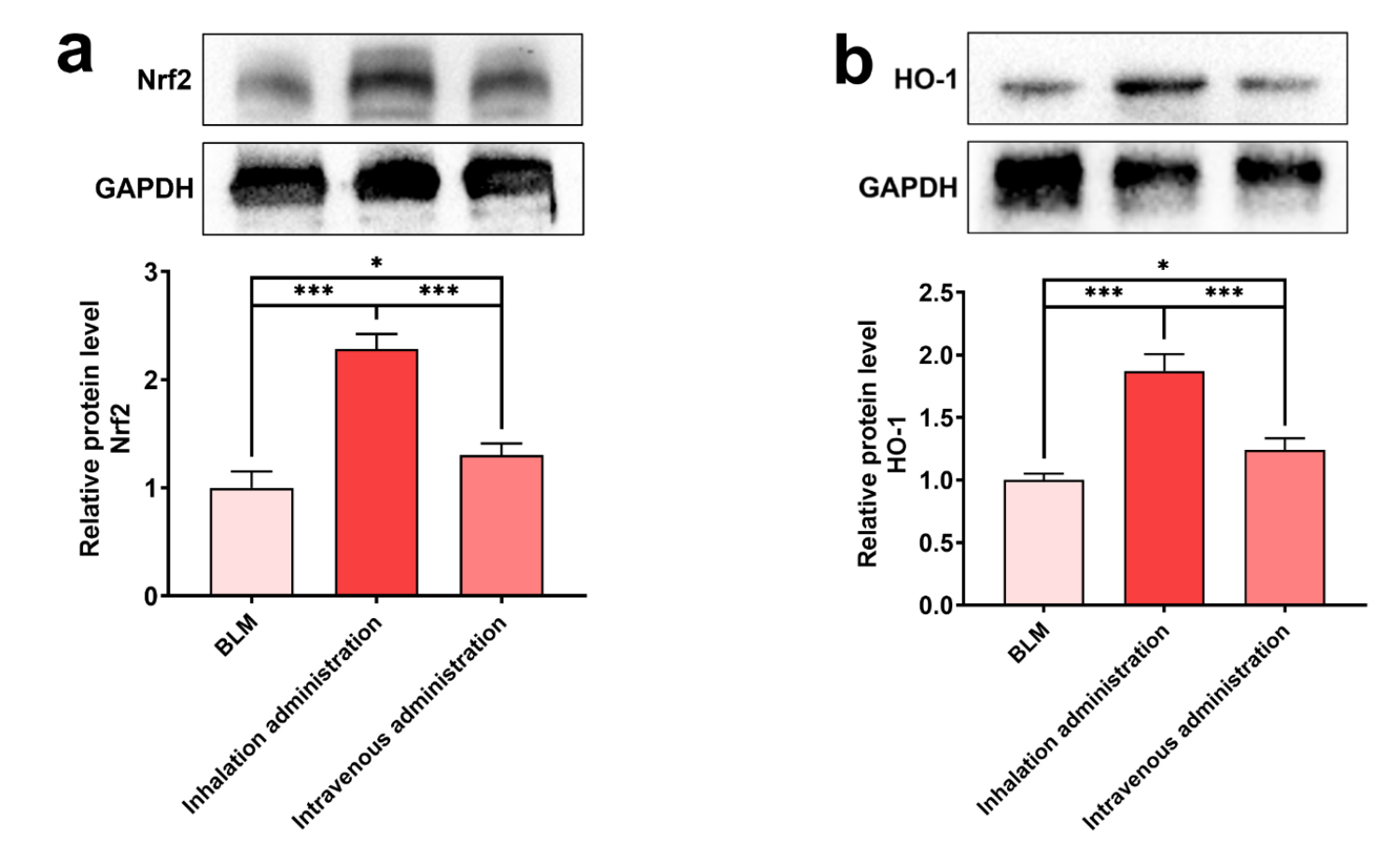

Supplement: Supplementary file 16 — Additional file 16: Fig. S16. The effects of DTP@DMF NPs inhalation and DMF intravenous administration on Nrf2 signaling in lung tissue. (a) The Nrf2 and (b) HO-1 expression in lung tissue (n = 3). * P < 0.05, ** P < 0.01, *** P < 0.001. [file 12951_2022_1435_MOESM16_ESM.jpg]
